# The Antioxidant Activity of Prenylflavonoids

**DOI:** 10.3390/molecules25030696

**Published:** 2020-02-06

**Authors:** Clementina M. M. Santos, Artur M. S. Silva

**Affiliations:** 1 Centro de Investigação de Montanha (CIMO), Instituto Politécnico de Bragança, Campus de Santa Apolónia, 5300-253 Bragança, Portugal; 2 QOPNA & LAQV-REQUIMTE, Department of Chemistry, University of Aveiro, 3810-193 Aveiro, Portugal

**Keywords:** ABTS assay, antioxidant, chelation studies, DPPH radical, flavonoids, FRAP, lipid peroxidation, natural products, prenyl, ROS

## Abstract

Prenylated flavonoids combine the flavonoid moiety and the lipophilic prenyl side-chain. A great number of derivatives belonging to the class of chalcones, flavones, flavanones, isoflavones and other complex structures possessing different prenylation patterns have been studied in the past two decades for their potential as antioxidant agents. In this review, current knowledge on the natural occurrence and structural characteristics of both natural and synthetic derivatives was compiled. An exhaustive survey on the methods used to evaluate the antioxidant potential of these prenylflavonoids and the main results obtained were also presented and discussed. Whenever possible, structure-activity relationships were explored.

## 1. Introduction

Flavonoids are oxygen heterocyclic compounds widespread throughout the plant kingdom. This class of secondary metabolites are responsible for the color and aroma of many flowers, fruits, medicinal plants and plant-derived beverages and play an important protective role in plants against different biotic and abiotic stresses. Flavonoids are also known for their nutritional value and positive therapeutic effects on humans and animals [1,2]. 

The basic structure of a flavonoid consists of a dibenzo-γ-pyrone framework and the degree of unsaturation and oxidation of the C ring defines the subclasses of this compounds such as chalcones, flavones, isoflavones and their dehydro derivatives. Different substitution patterns that includes hydroxyl, methyl, methoxyl, prenyl and glycosyl groups can be attached to the flavonoid unit, varying the number, type and position of the substituents [3]. 

Over the past two decades there have been an increasing number of reports on the isolation of prenylated flavonoids, belonging to the Leguminosae and Moraceae families, with some distribution among others such as Cannabaceae, Euphorbiaceae, Guttiferae, Rutaceae, Umbelliferae, etc. [4,5]. Barron and Ibrahim reviewed more than 700 prenylated flavonoids up to the end of 1994 [5] and Botta et al. compiled it from 1995 till 2004 [6]. Most of the prenylated flavonoids have been identified as chalcones, dihydrochalcones, flavones, flavanones, flavonols and isoflavones, being *C*-prenyl more common than *O*-prenyl derivatives. In addition, prenyl side-chains can include variations in the number of carbons, oxidation, dehydration, cyclization or reduction to give a huge array of compounds with an impressive antioxidant potential [4].

Along the manuscript, “prenyl” will be used a general term to identify prenyl/isopentenyl, geranyl and farnesyl side chains as well as furano or dimethylchromano derivatives. 

The beneficial effects of flavonoids appear to be related to the various biological and pharmacological activities as anti-inflammatory, antimicrobial, antioxidant, antitumor, estrogenic, and immunosuppressive properties, among others. According to the literature, prenylation of flavonoids can induce an increase in their bioactivities namely as antimicrobial and anticancer agents, however, a decrease in the bioavailability and plasma absorption is recorded, when compared to related non-prenylated derivatives [7,8,9,10,11].

The available information concerning the antioxidant activity of prenylated flavonoids is sparse, appearing some studies in a couple of review papers [7,8]. Taking into account our both interest (organic and medicinal chemistry) for the identification of prenylated flavonoids that has already been tested for their antioxidant activity, a systematic revision of the literature was made using PubMed^®^ and Web of Knowledge^®^ databases. The research was limited to the 21st century, with publications from January 2000 to October 2019, using the terms “prenyl”, “flavonoid”, “prenylated flavonoid” in combination with “antioxidant”, as keywords. After a careful analysis of nearly one hundred of papers, we excluded many of them due to the absence of the antioxidant effects for the target compounds, leaving 59 papers to be included and discussed in the present review.

Herein, it is our propose to organize and summarize the structural and chemical diversity of natural and synthetic flavonoids applied as antioxidants, providing the main in vitro and in vivo methodologies used in this field of research. This information is very useful for organic chemists to know the trends of structure-antioxidant activity relationship, in order to develop efficient routes for the total synthesis of natural derivatives, to develop improved strategies to maximize the synthesis of such natural and synthetic compounds or even in the design and synthesis of novel flavonoids with different prenyl substituents in different positions of the main skeleton. So, the studies here summarized and the promising results obtained highlights the importance of prenylated flavonoids as potential antioxidant agents.

## 2. Natural Occurrence and Structural Variation of Prenylflavonoids with Antioxidant Activity

From the natural prenylflavonoids with antioxidant activity most of them are from Moraceae and Fabaceae families with a limited number of derivatives from Apiaceae, Asteraceae, Cannabaceae, and Euphorbiaceae. 

**Figure 1:**
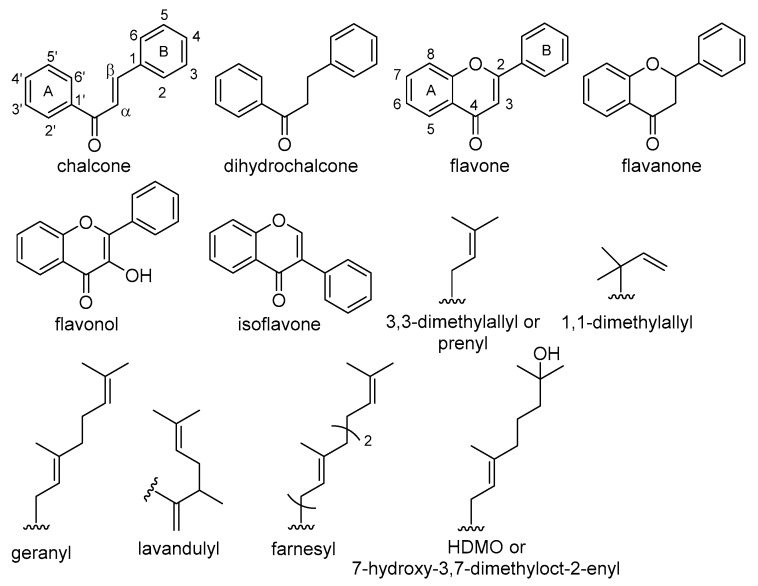
Chemical structures of some flavonoids and main prenylation patterns.

The most common substitution among the flavonoid family with antioxidant properties is represented by the 3,3-dimethylallyl chain. l,l-Dimethylallyl, geranyl, lavandulyl and farnesyl units can also be present in such structures. They can be found in chalcones, dihydrochalcones, flavones, flavanones, isoflavones, xanthone-type and other complex molecules (Figure 1). As referred before, *C*-prenylated compounds are more abundant than *O*-prenylated ones, being most of the *O*-prenylflavonoids obtained by synthesis.

Chalcone derivatives. This is the most abundant class of prenylated flavonoids with antioxidant activity, being a great number of the natural derivatives obtained from different extracts of hops [12,13,14,15,16,17], belonging to the Cannabaceae family. A limited number of derivatives can be obtained from Moraceae [18,19,20], Fabaceae [21,22,23] and Asteraceae [24] families. More than half of the chalcones, mainly derivatives of xanthohumol, were prepared by synthesis [12,13,14,25,26,27,28,29] (compounds **1**–**44**, Table 1). To refer that Popoola et al. identified isoxanthohumol isolated from the aerial part of *Helichrysum teretifolium* as a chalcone derivative **21** [24] while the remaining literature classify isoxanthohumol as a flavanone derivative **102** [12,13,14]. 

**Figure 2:**
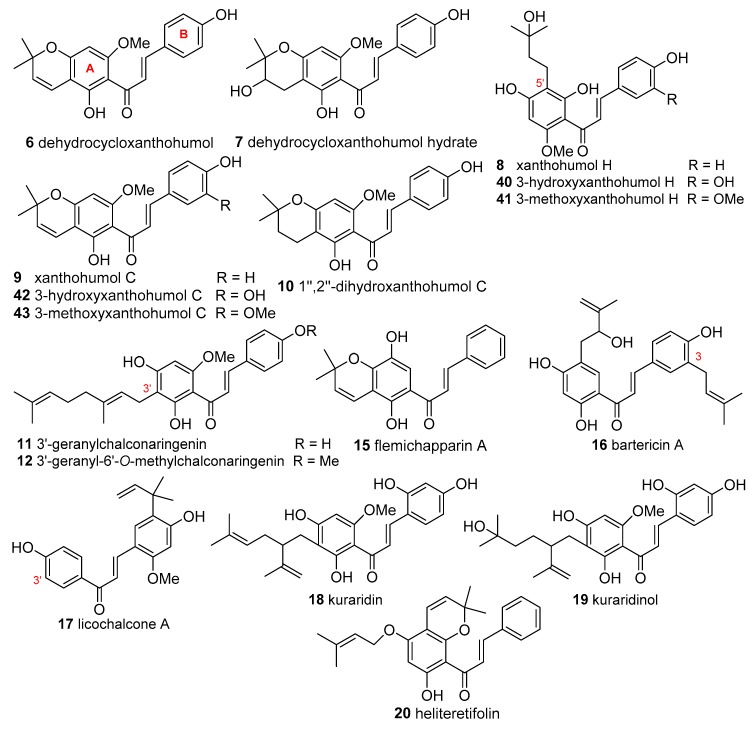
Chemical structures of other prenylated chalcones **6**–**12**, **15**–**20** and **40**–**43** with antioxidant activity.

**Figure 3:**
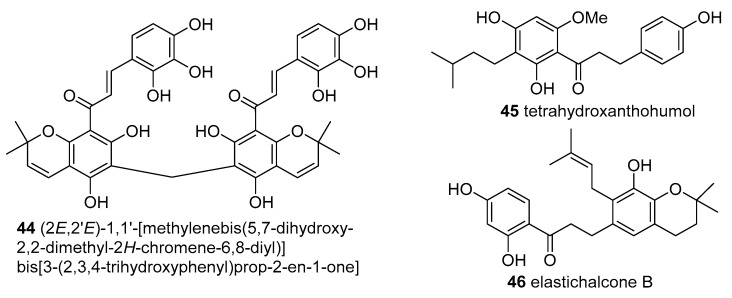
Chemical structures of chalcone dimer **44** and dihydrocalcones **45** and **46** with antioxidant activity.

Most of the known derivatives are prenylated on the A-ring (Table 2), except licochalcone A (**17**) that has an 1,1-dimethylallyl unit at C-5 of B-ring (Figure 2). Among the former ones, 3′-prenyl substitution is the most abundant side attachment, being most of these derivatives also hydroxylated at positions 2′ > 4 > 4′ and methylated at positions 6′ > 4′ > 2′ (Table 2). Other examples are those two having a 3′-geranyl group (compounds **11** and **12**), two 3′-lavandulyl derivatives (compounds **18** and **19**), three derivatives of xanthohumol H possessing a 3-hydroxy-3-methylbutyl group at C-5′ (compounds **8**, **40** and **41**) and bartericin A (**16**), an example of a diprenylated chalcone with a 3-prenyl and a 5′-(2-hydroxy-3-methylbut-3-enyl) moieties (Figure 2). The remaining chalcones exhibit a ring, appearing most of the times as 6,7-(2,2-dimethylchromeno) derivatives. Among them, the *O*-prenylated chalcone reported as heliteretifolin (**20, Figure 2**), isolated from a *H. teretifolium* methanolic extract and the chalcone dimer **44** (Figure 3) obtained by synthesis.

In contrast to the chalcones, only two prenylated dihydrochalcones, elastichalcone B (**46**) isolated from the leaves of *A. elasticus* [30] and tetrahydroxanthohumol (**45**) obtained by synthesis [12,13,14], are reported (Figure 3).

**Figure 4:**
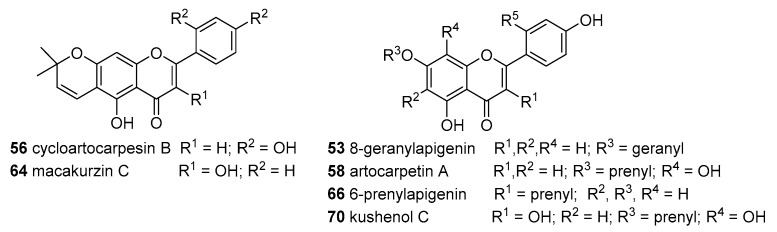
Chemical structures of mono-*C*-prenylated flavones **53**, **56**, **58**, **64**, **66** and **70** with antioxidant activity.

**Figure 5:**
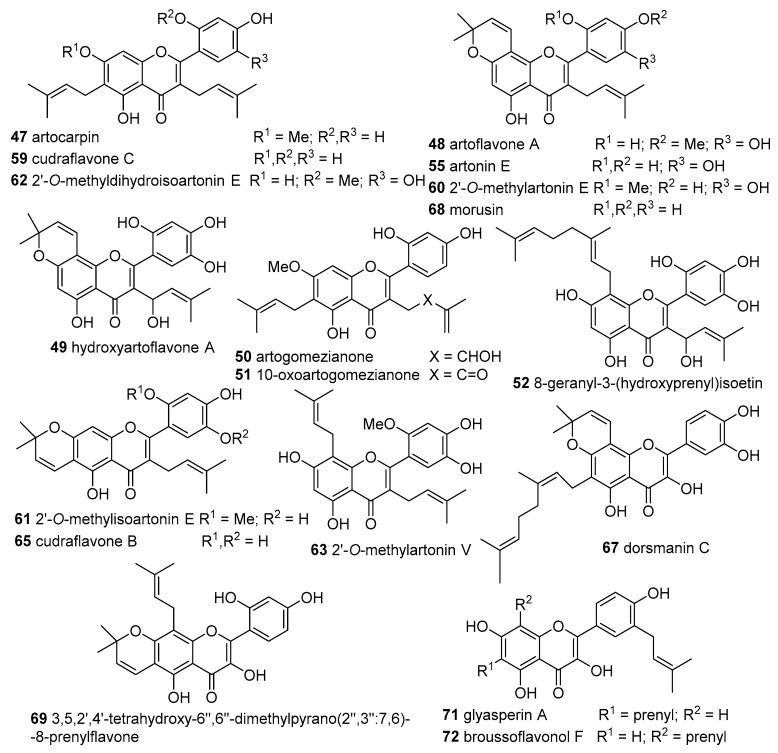
Chemical structures of di-*C*-prenylated flavones **47**–**52**, **55**, **59**, **60**–**63**, **65**, **67**–**69**, **71** and **72** with antioxidant activity.

**Figure 6:**
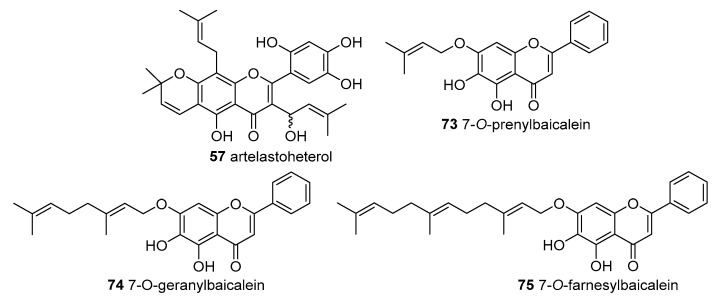
Chemical structures of triprenylated flavone **57** and mono-*O*-prenyated flavones **73**–**75** with antioxidant activity.

Flavone derivatives. The naturally-occurring derivatives were obtained mainly from Moraceae family, belonging to *Artocarpus* [18,19,21,30,31,32,33,34,35,36], *Dorstenia* [20,37] and *Cudrania* [38,39] species (compounds **47**–**76**, Table 3 and Figure 4, Figure 5 and Figure 6). A few examples from Fabaceae [22,40] and Euphorbieaceae [41,42] families were also isolated. Only three analogues were synthesized and were *O*-prenylated ones (7-*O*-prenyl- **73**, 7-*O*-geranyl- **74** and 7-*O*-farnesylbaicalein **75**) (Table 3 and Figure 6) [43].

All the natural prenylflavones with antioxidant activity are C-substituted, being most of them mono- (Figure 4) and di-prenylated (Figure 5). There is a single case of a triprenylated derivative, artelastoheterol (**57**), isolated from *Artocarpus elasticus* (Table 3 and Figure 6) [34].

Flavanone derivatives. The compounds belonging to this group are isolated mainly from plants of the Fabaceae family [22,23,40,44,45,46]. Several derivatives were also isolated from Moraceae [16,20,37,38,39], Asteraceae [24] and Cannabaceae [12,13,14] families. A great number of prenylated flavanones were collected from propolis of different origins [47,48] and some of them were obtained by synthesis [12,14,49] (compounds **77**–**118**, Table 4).

**Figure 7:**
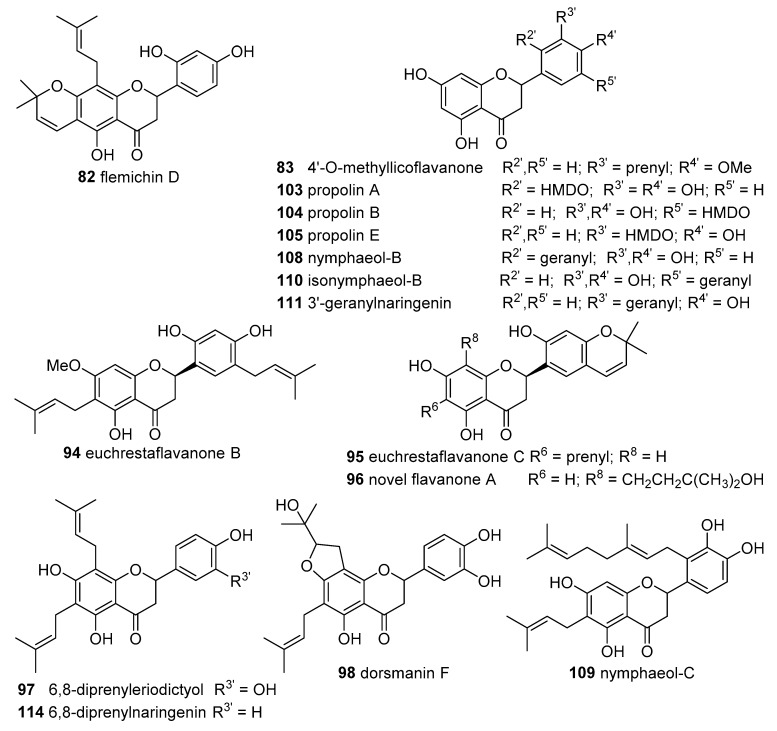
Chemical structures of flavanones diprenylated in A-ring **97**, **98** and **114**; monoprenylated in B-ring **83**, **103**–**105**, **108**, **110** and **111** and prenylated in A- and B-ring **94**–**96** and **109** with antioxidant activity.

**Figure 8:**
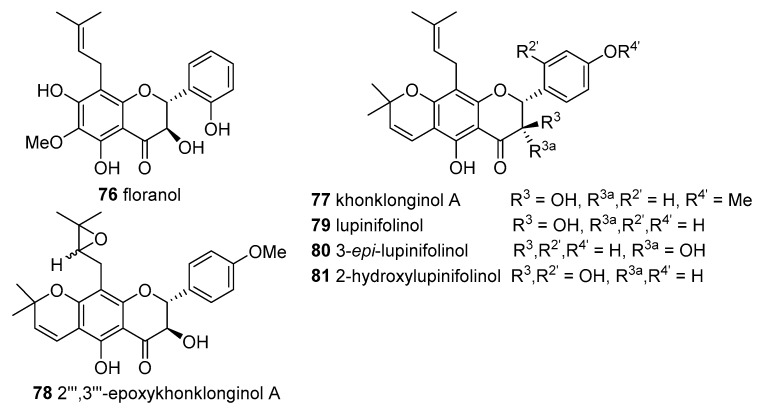
Chemical structures of prenylated flavanonols **76**–**81** with antioxidant activity.

Flavanones represent the second most abundant class of prenylated flavonoids with antioxidant activity. These derivatives can be grouped according to their substitution in the flavanone moiety. Thus, a great number of compounds are monoprenylated in A-ring (Table 5), although one can also find diprenylated in A-ring, monoprenylated in B-ring and prenylated in both A- and B-rings (Figure 7). Some prenylated flavanonols can also be found in Figure 8.

**Figure 9:**
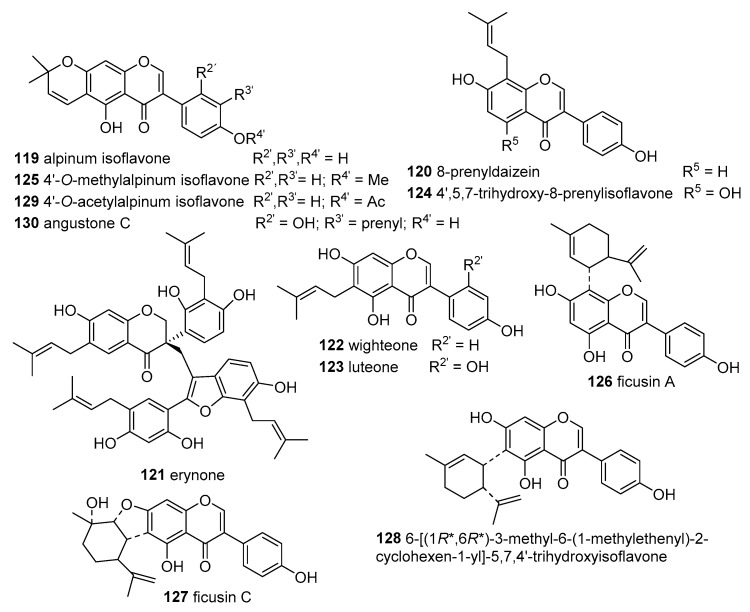
Chemical structures of prenylated isoflavones **119**–**130** with antioxidant activity.

Isoflavone derivatives. Prenylated isoflavones were isolated only from Fabaceae [46,50,51], Moraceae [52] and Apiaceae [53] families (compounds **119**–**130**, Table 6). The major part of the derivatives are prenylated in A-ring, as 6-prenyl or 8-prenyl, along with some derivatives with a 6,7-(2,2-dimethylchromene)-fused unit. A single example of isoflavone with a prenyl moiety in B-ring is angustone C (**130**), isolated from *Azorella madreporica*. Erynone (**121**) isolated from *Erythrina stricta*, represents a complex isoflavanone substituted with several prenyl units (Figure 9).

**Figure 10:**
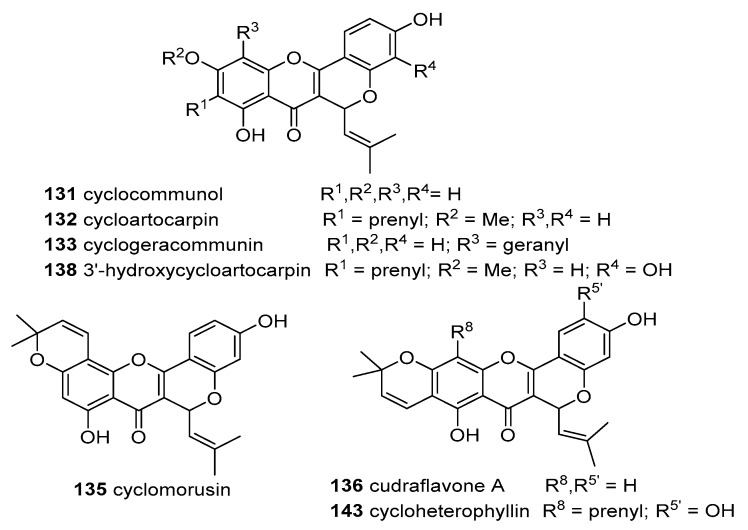
Chemical structures of prenylated xanthone-type derivatives **131**–**133**, **135**, **136**, **138** and **143** with antioxidant activity.

**Figure 11:**
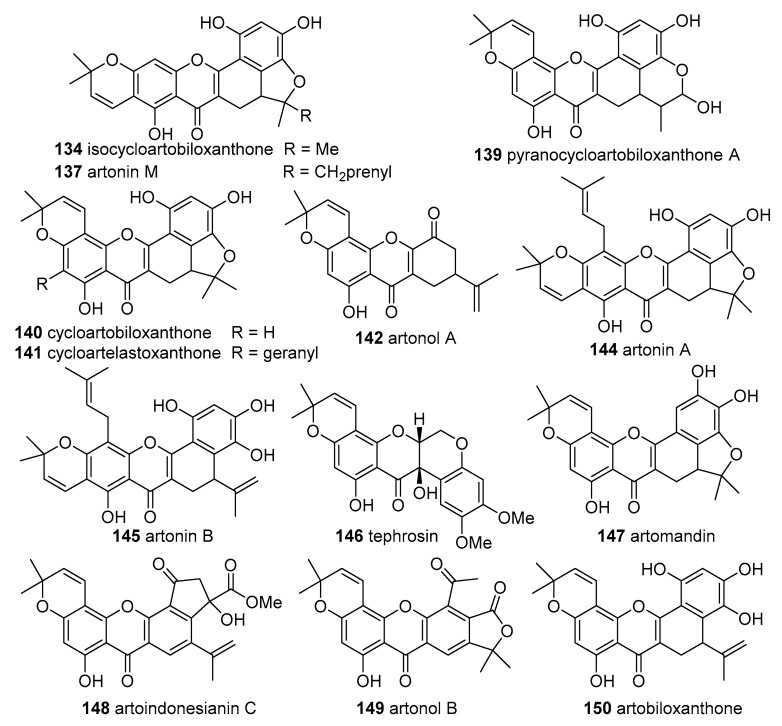
Chemical structures of prenylated xanthone-type derivatives **134**, **137**, **139**–**142** and **144**–**150** with antioxidant activity.

Xanthone-type compounds. All prenylated xanthone-type derivatives were isolated from different species of *Artocarpus*: *A. altilis* [35], *A. anisophyllus* [18], *A. communis* [34], *A. elasticus* [31], *A. heterophyllus* [12], *A. incisa* [32], *A. integer* [36], *A. kemando* [54], *A. nobilis* [33], *A. obtusus* [55], *A. scortechinii* [19], which belongs to Moraceae family (compounds **131**–**150**, Table 7). In this group we can find compounds with at least four-fused rings which structures may result from the cyclization of the prenyl group in the flavone unit to give a pyran ring (Figure 10) or can be xanthone nucleus, saturated or not, possessing other rings attached to the main core, leading to compounds with at most six-fused rings (Figure 11).

**Figure 12:**
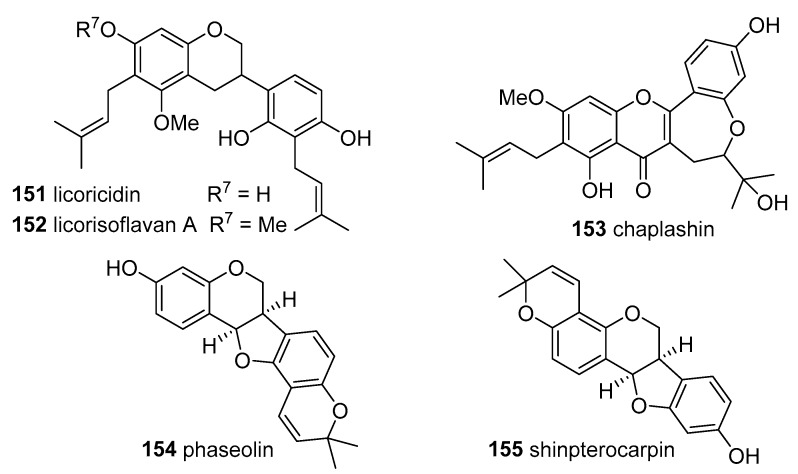
Chemical structures of prenylated flavonoid-type derivatives **151**–**155** with antioxidant activity.

Miscellaneous compounds. Only two examples of isoflavan-type, licoricidin (**151**) and licorisoflavan A (**152**), were isolated from *Glycyrrhiza uralensis* Fisher [21] (Figure 12). Chaplashin (**153**), a flavone containing an oxepin ring, was isolated for the first time from the leaves and the heartwoods of *A. anisophyllus* Miq [18]. Two prenylated pterocarpans, phaseollin (**154**) and shinpterocarpin (**155**), have been isolated from the stem bark of *Erythrina orientalis* [46] (Figure 12).

## 3. Methods for the Evaluation of the Antioxidant Activity of Prenylflavonoids

Various methods have been applied to study the antioxidant properties of a wide variety natural and synthetic prenylated flavonoids. For the in vitro methods, the most common ones are those involving electron transfer mechanisms such as DPPH, FRAP and TEAC assays; hydrogen atom transfer mechanisms such as for the inhibition of ROS and RNS scavenging assays and metal chelation studies. In the former case, DPPH radical scavenging method is by far the most frequently used, probably due to its simplicity in terms of time effort, experimental procedure and cheap reagents. Considering the in vivo models, two methods were used to evaluate the antioxidant potential of several prenylated flavonoids that include lipid peroxidation assay and LDL oxidation assay.

### 3.1. In Vitro Methods

#### 3.1.1. Electron Transfer Mechanisms

##### DPPH Radical Scavenging Activity

**Scheme 1:**
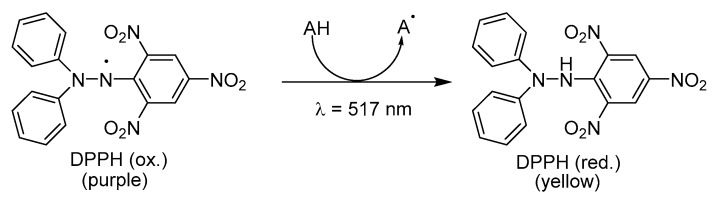
Reaction scheme involved in DPPH radical scavenging activity assay.

DPPH^●^ (1,1-diphenyl-2-picrylhydrazyl) is a stable free radical characterized by an absorption band at about 517 nm. In the presence of an antioxidant molecule (AH), DPPH^●^ trap a hydrogen atom to its reduced hydrazine form with consequent loss of the typical purple colour to a pale yellow one (Scheme 1).

The percentage of the DPPH^●^ scavenging is calculated according to the following Equation (1):

(1) $$\%\;\mathrm{inhibition}\;\mathrm{of}\;\mathrm{DPPH}\;\mathrm{radical}=\left(\frac{A_{\mathrm{control}}-A_{\mathrm{sample}}}{A_{\mathrm{control}}}\right)\times 100$$

where A_control_ is the absorbance of the control (before the reaction take place) and A_sample_ is the absorbance after the reaction occurred [56].

The activity is expressed as inhibitory concentration IC_50_, that is the amount of antioxidant necessary to decrease by 50% the initial DPPH^●^ concentration. Some disadvantages of this method is the steric accessibility of the radical by large antioxidant molecules, spectrophotometric measurements can be affected by compounds that absorb at the same wavelength of the determination and cannot be applied for measuring the antioxidant capacity of plasma since precipitation of proteins may occur in alcoholic media.

##### ABTS Radical Cation Scavenging Activity

**Scheme 2:**
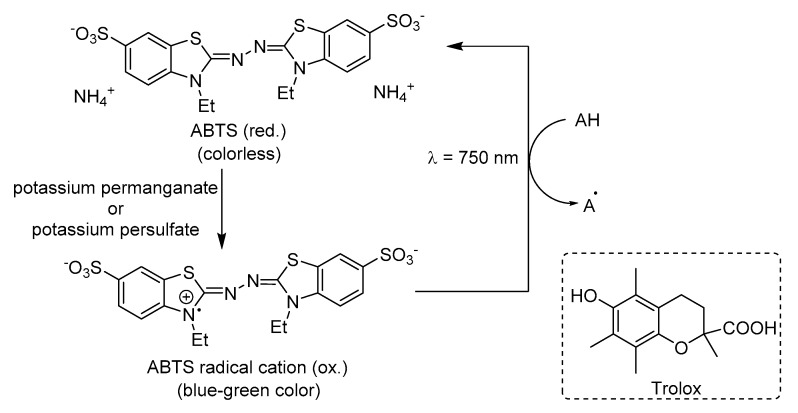
Reaction scheme involved in ABTS radical cation scavenging activity assay.

ABTS^●+^ (2,2-azino-bis(3-ethylbenzthiazoline-6-sulfonic acid) is a stable blue–green chromophore radical cation characterized by an absorption band at about 750 nm which losses its colour in the presence of an antioxidant molecule (Scheme 2). ABTS^●+^ is generated by reacting a strong oxidizing agent (e.g., potassium permanganate or potassium persulfate) with the ABTS salt. In this assay, Trolox (6-hydroxy-2,5,7,8-tetramethylchroman-2-carboxylic acid) is usually used as antioxidant standard. The obtained results are expressed as Trolox equivalent antioxidant capacity (TEAC) values from the trolox standard curve [56]. This methodology can be applied to measure both hydrophilic and lipophilic antioxidant capacities since the ABTS^●+^ is soluble in both aqueous and organic solvents and is not affected by ionic strength of the medium.

##### Ferric Reducing Antioxidant Power (FRAP) Method

**Scheme 3:**
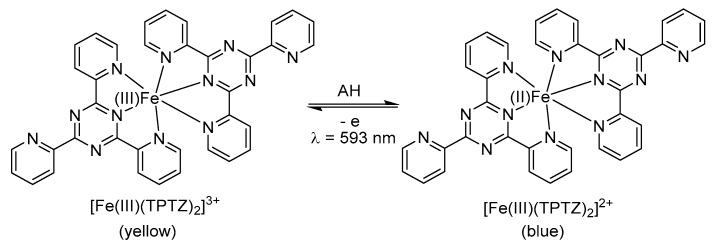
Reaction scheme involved in FRAP assay.

This method is based on the reduction of the colourless complex of ferric iron and 2,3,5-triphenyl-1,3,4-triaza-2-azoniacyclopenta-1,4-diene chloride (TPTZ) to the blue-coloured ferrous form at low pH (Scheme 3). FRAP reagent is produced by mixing acetate buffer (pH 3.6), TPTZ solution and FeCl_3_ 6H_2_O. FRAP values are obtained by comparing the absorbance change at 593 nm in reaction mixtures with those containing ferrous ions in known concentration [17,53]. The redox potential of Fe(III) salt (−0.70 V) is comparable to that of ABTS^●+^ (0.68 V), therefore, the main difference between TEAC assay and the FRAP assay is that the first is carried out at neutral pH and latest under acidic (pH 3.6) conditions. Usually a calibration curve using antioxidant trolox is made and the results are expressed as trolox equivalents per kg (solid food) or per L (beverages) of sample [56].

#### 3.1.2. Hydrogen Atom Transfer Mechanisms

##### Superoxide Radical Anion Scavenging Activity

**Scheme 4:**
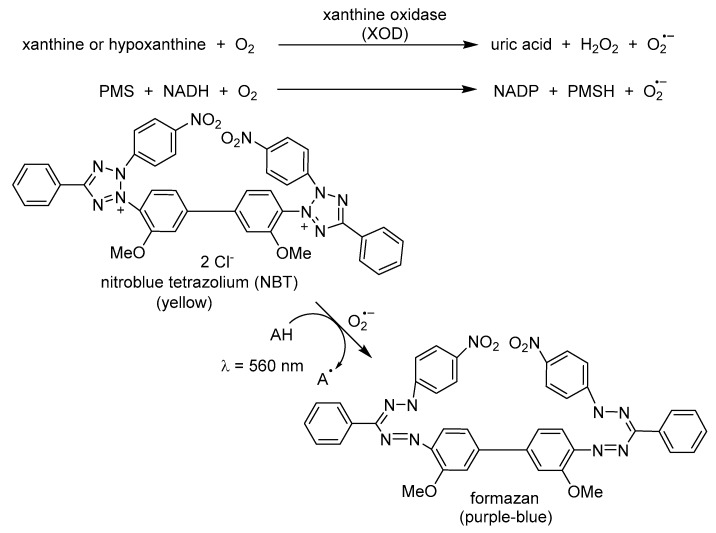
Reaction scheme involved in superoxide radical anion scavenging activity assay.

Superoxide radical anion (O_2_^•−^) can be generated by two different approaches, using hypoxanthine or xanthine/xanthine oxidase (XOD) system at pH 7.4 [14,26,35] or using a non-enzymatic reaction of phenazine methosulphate (PMS) in the presence of nicotinamide adenine dinucleotide (NADH) [56] (Scheme 4). In both systems, superoxide anion radicals can reduce nitroblue tetrazolium (NBT) into formazan, and the effects are determined spectrophotometrically at 560 nm. Higher is the scavenging potential of the antioxidant molecule, lower is the formation of formazan and consequently, lower is the absorbance [56]. Other detectors can be used, being cytochrome c the second option, which reduction is followed spectrophotometrically at 550 nm [31,32]. The results are typically expressed as inhibitory concentration IC_50_ values.

##### Oxygen Radical Absorbance Capacity (ORAC) Method

The ORAC method can be applied to both hydrophilic and lipophilic environments and for the detection of both hydroxyl and peroxyl radicals, formed during lipid oxidation chain reactions (autoxidation) and involving hydrogen atom transfer reactions.

**Scheme 5:**
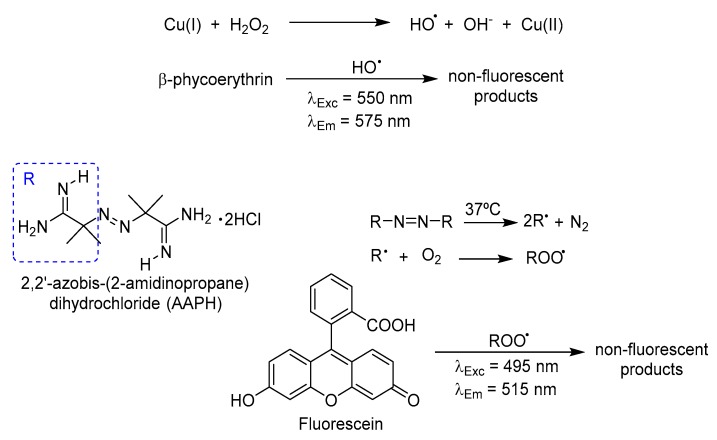
Reaction schemes involved in ORAC assay for the detection of hydroxyl and peroxyl radicals.

The system H_2_O_2_-CuSO_4_ is generally used as hydroxyl radical generator and β-phycoerythrin used as a redox-sensitive fluorescent indicator protein, which decay in the fluorescence is measured in the presence of free radical scavengers, using Trolox as standard (Scheme 5). The ORAC value is then calculated from the trolox equivalent and expressed as ORAC units or value by taking the difference of areas-under-the-decay curves between blank and sample and/or standard. Higher the ORAC value, higher the antioxidant potential of the tested compounds [56].

2,2′-Azobis-(2-amidinopropane)dihydrochloride (AAPH) is the most used peroxyl radical generator in hydrophilic systems [24] and as fluorescent probe can be used β-phycoerythrin or more recently fluorescein [16,25,28]. Thus, peroxyl radical are formed by thermodecomposition of AAPH, giving an alkyl radical that react with molecular oxygen to give peroxyl radical. The decay in fluorescence is recorded and the results of the scavenging activity is expressed as Trolox equivalents [56].

##### Other ROS/RNS Scavenging Activity

Other methodologies can be applied to evaluate the antioxidant potential of a series of natural and synthetic matrix against a series of reactive oxygen and nitrogen species (ROS and RNS). For prenylated flavonoids there are only a couple of papers referring the scavenging activity profile against hydrogen peroxide [31] and peroxynitrite anion [22], which led us to describe them in the next section, prior the discussion of the results obtained in such assays.

#### 3.1.3. Metal Chelation

Copper and iron chelation by prenylated flavonoids were determined by the difference in thein the UV-vis spectra (190–900 nm) produced when this metal ions were incubated with the tested compounds. The results are expressed as the difference in the absorbance or spectral shift of sample in the presence and in the absence of the metal ions [31,44,56].

### 3.2. In Vivo Methods

#### 3.2.1. Lipid Peroxidation Assay

Lipid peroxidation is usually induced by metal ions such as iron and measured by the thiobarbituric acid method, being the levels of peroxides formed expressed as TBARS. Other systems used for prenylated flavonoids were Mb(IV)-induced arachidonic acid peroxidation [32] and metal-ion independent systems using *tert*-butyl hydroperoxide (TBHP)-induced lipid peroxidation in liver microsomes or oxidation of β-carotene/linoleic acid emulsion. One of the limitations of this technique is the time consuming that depends on the oxidation of a substrate which is influenced by temperature, pressure, matrix, etc., particularly important when a great numbers of samples are involved. [47].

#### 3.2.2. LDL Oxidation Assay

Cu(II)-induced low-density lipoprotein (LDL) oxidation is determined by measuring, in an initial stage, the formation of conjugated dienes through the increase of absorbance at 234–250 nm and at the end, the generated amount of lipid peroxides by the TBARS assay at 532 nm, using MDA for the standard curve [12]. 3-Morpholinosydnomine (SIN-1), a peroxynitrite generator, can also be used to induce LDL oxidation [15].

## 4. Antioxidant Effects of Prenylflavonoids

### 4.1. In Vitro Methods

#### 4.1.1. Electron Transfer Mechanisms

##### DPPH Radical Scavenging Activity

A great number of prenylated flavonoid derivatives were evaluated for their ability to scavenge DPPH^●^. Ko et al. evaluated DPPH^●^ decolorization induced by xanthone-type cycloheterophyllin (**143**), artonin A (**144**) and artonin B (**145**), isolated from the plant *A. heterophyllus* Lam. The scavenging activity was expressed as the concentration (IC_0.20_) of the test compounds that induced a decrease of 0.20 in absorbance in a 30 min period of time. The results pointed out that **143**–**145** increased DPPH^●^ decolorization in a concentration-dependent manner with IC_0.20_ of 9.6 ± 0.7, 8.4 ± 0.3, and 12.2 ± 0.6 μM, respectively. The positive control α -tocopherol scavenged DPPH^●^ with an IC_0.20_ of 11.9 ± 0.2 μM [31].

The flavanones 6,8-diprenyleriodictyol (**97**) and dorsmanin F (**98**) and the flavonol dorsmanin C (**67**) were isolated from the twig and leaf of *D. mannii* samples collected in Central Province of Cameroon. The DPPH^●^ scavenging activity assay was performed in experiments of only 20 min since a plateau at 15 min was reached. The prenylated flavonoids were tested in concentrations of 1, 10 and 100 μM, causing a rapid decrease in the absorbance, dependent of the concentration, and compared with butylated hydroxytoluene (BHT), a common antioxidant used as a food additive. The potency of DPPH^●^ scavenging activity followed the order: **67** > **97** > **98** >> BHT. These results seem to indicate that the C2=C3 double bond and the 3-OH group of the flavonol are important features for the high scavenging potency, when compared with the flavanones [37].

Two flavanones, propolin A (**103**) and propolin B (**104**), were isolated and characterized from Taiwanese propolis glue collected from hives located in the area of Bagwa Shan, Taiwan. Both compounds were tested in concentrations ranging from 3.125 to 25 μg/mL and exhibited strong scavenging effects against DPPH^●^ with IC_50_ values of 5.0 and 9.0 μg/mL, respectively [48].

Omisore et al. tested the DPPH^●^ scavenging effects of five flavonoids from *Dorstenia* species: the chalcones isobavachalcone (**14**) and bartericin A (**16**) isolated from *D. barteri*, the flavone 6-prenyl-apigenin (**66**) from *D. kameruniana*, and the flavanones 6,8-diprenyleriodictyol (**97**) and dorsmanin F (**98**) isolated from *D. mannii*. The concentration needed to decrease the remaining DPPH^●^ by 50% (the initial substrate concentration EC_50_) was the parameter used to measure the antioxidant capacity. Thus bartericin A (**16**, EC_50_ 47.85 ± 2.15 μg/mL) and 6,8-diprenyleriodictyol (**97**, EC_50_ 32.12 ± 1.10 μg/mL) as well as the positive controls quercitrin (EC_50_ 28.16 ± 0.84 μg/mL) and ascorbic acid (EC_50_ 19.33 ± 0.3 μg/mL) showed high antioxidant capacity (EC_50_ < 50 μg/mL), while isobavachalcone (**14**, EC_50_ 84.33 ± 0.27 μg/mL), 6-prenylapigenin (**66**, EC_50_ 86.43 ± 0.26 μg/mL) and dorsmanin F (**98**, EC_50_ 53.89 μg/mL) presented moderate antioxidant capacity (EC_50_ > 50 μg/mL). The scavenging effects followed the order: ascorbic acid > quercitrin > 6,8-diprenyleriodictyol (**97**) > bartericin A (**16)** > dorsmanin F (**98)** > isobavachalcone (**14**) > 6-prenylapigenin (**66**) [20]. A detailed overview on the natural occurrence, synthesis, biosynthesis and pharmacological properties of isobavachalcone (**14**) was published by Kuete and Sandjo in 2012 [57].

From the root bark of *Cudrania tricuspidata* (Carr.) Bureau collected in Hyoupchun, Korea it was possible to isolate two flavones—cycloartocarpesin B (**56**) and cudraflavone B (**65**)—and three flavanones—euchrestaflavanone B (**94**), euchrestaflavanone C (**95**) and a novel flavanone A (**96**). None of the isolated flavonoids were effective against DPPH^●^, presenting only 10% scavenging activity at 300 μM concentration [38].

Kumazawa et al. isolated nine flavanones (propolin A (**103**), propolin B (**104**), prokinawan (**106**), propolin E (**105**), nymphaeol A (**107**), nymphaeol B (**108**), nymphaeol C (**109**), isonymphaeol B (**110**) and 3′-geranylnaringenin (**111**)) from propolis collected in Okinawa, Japan. The antioxidant properties against DPPH^●^ at concentrations ranging from 3.125 to 100 μM, after 1 h of incubation were examined. BHT, α-tocopherol, and eriodictyol were tested as positive controls. A strong DPPH^●^ scavenging activity was recorded for compounds **103**, **104** and **106**–**110** with IC_50_ values between 5.2 and 10.9 μM, similar to those obtained for the positive controls BHT (IC_50_ 16.8 ± 2.7 μM), α-tocopherol (IC_50_ 11.4 ± 0.9 μM) and eriodictyol (IC_50_ 4.7 ± 0.7 μM). Propolin E (**105**) and 3′-geranylnaringenin (**111**) showed IC_50_ values of 62.6 ± 2.2 and 64.2 ± 3.5 μM, respectively. The high scavenging effects of these compounds may be related to the catechol unit present in such structures, an important characteristic for the antioxidant activity of flavonoids [47].

Five flavones—artonin E (**55**), 2′-*O*-methylartonin E (**60**), 2′-*O*-methylisoartonin E (**61**), 2′-*O*-methyldihydroisoartonin E (**62**) and 2′-*O*-methylartonin V (**63**)—and two xanthone-type compounds—artobiloxanthone (**150**) and cycloartobiloxanthone (**140**)—were obtained from the root bark of *A. nobilis* collected in Central Province of Sri Lanka. The authors claimed to have evaluated the DPPH^●^ scavenging potential by a TLC bio-autography method and that all the compounds were strong scavengers, but in fact no data was published in the manuscript [33].

Jung et al. isolated from *Sophora flavescens* collected in Kyeong Buk Province, Korea eight flavonoids: the chalcones kuraridin (**18**) and kuraridinol (**19**), the flavonol kushenol C (**70**) and the flavanones leachianone (**89**), kushenol E (**90**,), sophoraflavanone G (**91**), kurarinone (**92**) and kurarinol (**93**). The DPPH^●^ decolorization was investigated at 520 nm after 30 min of reaction. The results pointed out that flavanones **89**–**93** were ineffective scavengers, with percentages of inhibition less than 50 at a concentration of 200 μg/mL. On the other hand, flavonol **70** showed the highest scavenging activity with an IC_50_ value of 10.67 ± 0.23 μM, similar to that of the positive control, L-ascorbic acid (IC_50_ 8.70 ± 0.22 μM) followed by chalcone **19** and chalcone **18** with IC_50_ values of 86.23 ± 2.44 and 111.77 ± 0.72 μM, respectively [22]. Once again, the high scavenging activity of flavonol compared to flavanones can to be related to the presence the C2=C3 double bond and the 3-OH group in the flavonol skeleton. In another study, sophoraflavanone G (**91**) and kurarinone (**92**) exhibited IC_50_ values of 5.26 and 7.73 μg/mL, respectively, in DPPH^●^ scavenging assay, with no mention to positive control data [23].

Six derivatives were isolated from *Artocapus* species and their DPPH^●^ scavenging activity investigated. The flavone artoflavone A (**48**) and the xanthone-type compound cyclogeracommunin (**133**) were isolated from the cortex of roots of *A. communis* collected at Kaohsiung Hsien, Taiwan [34]. The flavone artelastoheterol (**57**) and the xanthone-type compounds cycloartobiloxanthone (**140**), cycloartelastoxanthone (**141**) and artonol A (**142**) were isolated from root bark of *A. elasticus* collected at Ping-Tung Hsien, Taiwan [58]. Cyclogeracommunin (**133**) and artonol A (**142**) were not able to scavenge DPPH^●^. Artoflavone A (**48**), artelastoheterol (**57**), cycloartobiloxanthone (**140**) and cycloartelastoxanthone (**141**) exhibited scavenging activity in a concentration-dependent manner with IC_50_ values of 24.2 ± 0.8, 42.2 ± 2.8, 26.8 ± 1.2 and 18.7 ± 2.2 μM, respectively. In addition, the IC_50_ values of the positive controls BHT and α-tocopherol were 80.0 ± 10.9 and 18.1 ± 1.5 μM, respectively. From the results we can state that compounds with a 2,2-dimethylpyran ring substituted at C-7 and C-8 of the flavonoid, such as derivatives **48**, **140** and **141**, enhanced the DPPH^●^ scavenging activity of the prenylated flavonoids [34].

Pyranocycloartobiloxanthone A (**139**) was isolated from the stem bark of the endemic and rare *A. obtusus* collected from Sarawak, Malaysia and showed strong DPPH^●^ scavenging activity with an IC_50_ value of 2.0 μg/mL. No information was given for the positive control [55].

Rahman et al. isolated two prenylated isoflavones from the stem bark of *E. variegata* collected at Dhaka, Bangladesh and evaluated their DPPH^●^ scavenging potential. 4′,5,7-Trihydroxy-8-prenyl isoflavone (**124**) and alpinum isoflavone (**119**) demonstrated high antioxidant activity, having IC_50_ values of 6.42 ± 1.36 and 8.30 ± 1.41 μg/mL, respectively, similar to that obtained by the positive control, *tert*-butyl-1-hydroxytoluene (BHT, IC_50_ 5.88 μg/mL) [51]. Very recently, a pharmacological overview on alpinum isoflavone (**119**) has been published by Ateba et al. [59].

Three xanthone-type derivatives were isolated from the stem bark of *A. kemando*: artomandin (**147**), artoindonesianin C (**148**) and artonol B (**149**). Although **147** scavenged DPPH^●^ with an IC_50_ of 38.0 μg 6.4 μg/mL, it was considerably lower effect than the positive control vitamin C (IC_50_ 12.2 μg/mL). In addition, artoindonesianin C (**148**) and artonol B (**149**) were weak scavengers, with IC_50_ values above 120 μg/mL [54].

5,7-Dihydroxy-6-methyl-8-prenylflavanone (**84**) 5,7-dihydroxy-4′-methoxy-6-methyl-8-prenyl-flavanone (**85**), 5,7-dihydroxy-6-prenylflavanone (**86**), 5-dihydroxy-7-methoxy-6-prenylflavanone (**87**), and 5,7-dihydroxy-4′-methoxy-8-prenylflavanone (**88**) were isolated from leaves of *E. platycarpa*. The scavenging effects were determined using concentrations of 10, 100 and 1000 μM of each compound and the results showed that the scavenging potential is directly proportional to the concentration of the prenylated flavanones. Moreover, flavanones did not show a remarkable reduction of DPPH^●^ being flavanone **88** the most active, with a 43.1 ± 3.9% of reduction for a 1000 μM concentration (the positive control quercetin presented 92.9% of reduction) [45].

Lan et al. isolated 14 derivatives from the heartwood and cortex of *A. altilis* and determined their DPPH scavenging effects. Seven flavones—artocarpin (**47**), artoflavone A (**48**), hydroxyartoflavone A (**49**), artogomezianone (**50**), 10-oxoartogomezianone (**51**), 8-geranyl-3-(hydroxyprenyl)isoetin (**52**), 8-geranylapigenin (**53**)—and seven xanthone-type compounds—cyclocommunol (**131**), cyclo-artocarpin (**132**), cyclogeracommunin (**133**), isocycloartobiloxanthone (**134**), cyclomorusin (**135**), cudraflavone A (**136**) and artonin M (**137**). Most of the tested compounds presented a weak scavenging effect with IC_50_ > 300 μM, except for hydroxyartoflavone A (**49**, IC_50_ 20.9 ± 2.1 μM) > isocycloartobiloxanthone (**134**, IC_50_ 33.9 ± 1.5 μM) > artoflavone A (**48** (15), IC_50_ 53.5 ± 3.1 μM). Even so, these compounds are weaker scavengers than the positive control, quercetin (IC_50_ 10.2 ± 1.4 μM) [35].

From the roots of *E. chinense* collected at Ubonratchathani Province, Thailand, was isolated one flavonol 3,5,2′,4′-tetrahydroxy-6″,6″-dimethylpyrano(2″,3″:7,6)-8-(3‴,3‴-dimethylallyl)flavone (**69**) and six flavanones—khonklonginol A (**77**), 2‴,3‴-epoxykhonklonginol A (**78**), lupinifolinol (**79**), 3-*epi*-lupinifolinol (**80**), 2-hydroxylupinifolinol (**81**) and flemichin D (**82**)—and their antioxidant activity was investigated using the DPPH^●^ scavenging system. Flavonol **69** was the most active compound showing an IC_50_ value of 35 ± 1 μM, similar to the obtained for BHT (IC_50_ 39 ± 1 μM). The order of efficacy for the remaining compounds were: **81** (252 ± 1.1) > **82** (538 ± 24) > **80** (681 ± 11) > **79** (1768 ± 210) > **78** (2553 ± 207) > **77** (7919 ± 55) μM [40].

Elastichalcone B (**45**) and cycloartocarpesin B (**56**) 3 isolated from the leaves of *A. elasticus* collected from Ulu Langat, Malaysia exhibited DPPH^●^ scavenging activity with IC_50_ values of 11.30 and 11.89 μg/mL, respectively. No data concerning any positive control was given by the authors [30].

Three isoflavones were isolated from the aerial parts of *Azorella madreporica* collected in Chile: alpinum isoflavone (**119**), angustone C (**130**) and 4′-acetylalpinum isoflavone (**129**). These compounds demonstrated modest DPPH^●^ scavenging activity in the following order: **130** (IC_50_ 134.61 ± 0.67 μM) > **119** (IC_50_ 160.75 ± 0.41 μM) > **129** (IC_50_ 309.87 ± 0.90 μM). Quercetin was used as positive control and reached an IC_50_ value of 24.93 ± 0.03 μM [53].

The chalcone xanthohumol (**1**) extracted from hop pellets (*Humulus lupulus*) did not show any scavenging effect on DPPH^●^ scavenging system [17]. One chalcone isobavachalcone (**14**), two flavones—4′,5-dihydroxy-6,7-(2,2-dimethylpyrano)-2′-methoxy-8-(γ,γ-dimethyl)allylflavone (**54**) and artocarpin (**47**)—one flavanone: 5,7-dihydroxy-4′-methoxy-8-prenylflavanone (**88**), three xanthones—3′-hydroxycycloartocarpin (**138**), pyranocycloartobiloxanthone A (**139**) and cycloartocarpin (**132**)—and the miscellaneous derivative chaplashin (**153**) were isolated for the first time from the leaves and the heartwoods of *A. anisophyllus* Miq collected in Malasya. Pyranocycloartobiloxanthone A (**139**), artocarpin (**47**) and 3′-hydroxycycloartocarpin (**138**) were the most active DPPH^●^ scavengers with SC_50_ (concentration required to produce 50% stimulation) values of 20.2, 140.0 and 152.9 μg/mL, respectively. Isobavachalcone (**14**), flavone **54** and chaplashin (**153**) were poor scavengers with SC_50_ values superior to 400 μg/mL and no scavenging activity was recorded for flavanone **88** and cycloartocarpin (**132**). BHA was used as positive control, with a SC_50_ value of 17.5 μg/mL [18].

Two prenylated pterocarpans—phaseollin (**154**) and shinpterocarpin (**155**)—the flavanone 4′-*O*-methyllicoflavanone (**83**) and two isoflavones, alpinum isoflavone (**119**) and 8-prenyldaidzein (**120**) have been isolated from the stem bark of *E. orientalis* collected in East Java, Indonesia. The results pointed out that antioxidant activity against DPPH^●^ scavenging by phaseollin (**154**, IC_50_ 241.9 μM) and 8-prenyldaidzein (**120**, IC_50_ 174.2 μM) were more effective than the positive control, ascorbic acid (IC_50_ 329.0 μM). The remaining compounds **83**, **119** and **155** showed moderate activity with IC_50_ values of 648.1, 708.5 and 909.8 μM, respectively [46].

Akter et al. isolated from stem bark of *E. stricta* Roxb., collected in India, four isoflavones—alpinum isoflavone (**119**), erynone (**121**), wighteone (**122**) and luteone (**123**)—and accessed their antioxidant activity using dot-lot and DPPH^●^ staining methods. Erynone (**121**) was the most active compound while luteone (**123**) possessed moderate antioxidant activity. Derivatives **122** and **123** were completely inactive against DPPH^●^ scavenging activity. The authors suggested that the high effect of erynone (**121**) is due to its highest number (six) of free phenolic hydroxyl groups and therefore high ability to donate phenolic hydrogens to DPPH^●^, when compared to the related structures **119**, **122** and **123** [50].

Stompor et al. synthesized isoxanthohumol (**102**) and two acetylated derivatives **117** and **118** and evaluated their DPPH^●^ scavenging properties. Acylation of **102** decreased the antioxidant activity of its analogues in the order: isoxanthohumol (**102**) > 4′-*O*-acetylisoxanthohumol (**117**) > 7,4′-di-*O*-acetylisoxanthohumol (**118**). Thus, **102** (EC_50_ 7.6 mM) is about 8-fold stronger antioxidant than its monoacyl derivative **117** (EC_50_ 59.7 mM) and 10-fold stronger than the diacyl derivative **118** (EC_50_ 73.5 mM) due to the presence of two free hydroxyl groups in the molecule [49].

A series of eight chalcones were obtained by synthesis: xanthohumol (**1**), 4-*O*-acetyl-xanthohumol (**25**), 4,4′-di-*O*-acetylxanthohumol (**26**), 4-*O*-decanoylxanthohumol (**27**), 4-*O*-dodecanoylxanthohumol (**28**), 4,4′-di-*O*-dodecanoylxanthohumol (**29**), 4-*O*-pivaloylxanthohumol (**30**) and 4,4′-di-*O*-pivaloylxanthohumol (**31**). All the compounds were tested for their ability to scavenge DPPH^●^ and the results expressed as Trolox equivalents, defined as the concentration of Trolox (μM) having the same activity as 1 g of the tested compound. Thus, the most active compound was **25** (15 μM TEAC/g), a two-fold stronger scavenger than **31** (37 μM TEAC/g) and **1** (38 μM TEAC/g). The values for the remaining compounds **25-30** ranged from 44 to 70 μM TEAC/g [27].

From the leaves and stem barks of *A. scortechinii* King collected at Pahang, Malaysia one chalcone flemichapparin A (**15**), four flavones—artocarpin (**47**), 4′,5-dihydroxy-6,7-(2,2-dimethylpyrano)-2′-methoxy-8-(γ,γ-dimethyl)allyflavone (**54**), artonin E (**55**) and macakurzin C (**64**)—and two xanthones—cudraflavone A (**136**) and cycloartobiloxanthone (**140**)—were isolated and their DPPH^●^ scavenging activity evaluated. It was not possible to determine the IC_50_ value of compounds **47**, **54**, **64** and **136** while derivatives **15**, **55** and **140** reached IC_50_ values of 131.0, 151.6 and 196.0 μM, respectively. Two positive controls were used in this assay being BHA a stronger scavenger (IC_50_ 49.87 μM), better than the analyzed compounds, and BHT considerably weaker, with an IC_50_ value of 231.6 μM [19].

Zakaria et al. isolated from heartwood of *A. integer* (Thunb.) Merr., collected in Indonesia, the flavones cudraflavone C (**59**) and artocarpin (**47**) and the xanthone tephrosin (**146**) and subjected them to the DPPH^●^ decoloration method at λ_max_ 500 nm. The flavones **59** and **47** were the most active, with IC_50_ values of 3.35 μg/mL and 4.70 μg/mL, respectively, while the xanthone **146** was 10-fold weaker with an IC_50_ value of 55.58 μg/mL. Ascorbic acid (IC_50_ 2.79 μg/mL) was used as positive control [36].

The rhizomes of *Ficus tikoua* collected in Yunnan Province, China, furnished five isoflavones—alpinum isoflavone (**119**), 4′-*O*-methylalpinum isoflavone (**125**), ficusin A (**126**), ficusin C (**127**) and 6-[(1R*,6R*)-3-methyl-6-(1-methylethenyl)-2-cyclohexen-1-yl]-5,7,4′-trihydroxyisoflavone (**128**)—and their ability to scavenge DPPH^●^ was investigated. All compounds exhibited moderate antioxidant activity with the EC_50_ values of 54.8 ± 9.7, 83.6 ± 12.5, 42.4 ± 6.6, 49.3 ± 7.8 and 43.3 ± 6.9 μM, respectively. Propyl gallate used as positive control presented an EC_50_ value of 1.8 ± 0.5 μM [52].

From the leaves of *Macaranga pruinosa* collected in Samarinda, Indonesia the flavone glyasperin A (**71**) was isolated and its antioxidant potential tested using a DPPH^●^ scavenging system. The IC_50_ value of **71** (443.0 ± 8.0 μg/mL) was almost twice higher than that of the positive control, kaempferol (IC_50_ 238.0 ± 3.3 μg/mL) [41].

From *Macaranga gigantea* (Euphorbiaceae) leaves collected in Indonesia the flavones glyasperin A (**71**) and broussoflavonol F (**72**) were isolated. The antioxidant activity was evaluated by their ability to scavenge DPPH^●^, showing IC_50_ values of 125.10 and 708.54 μM, respectively. In addition, the results pointed out that **71** was twice as active as the positive control ascorbic acid (329.01 μM) [42].

##### ABTS Radical Cation Scavenging Activity (TEAC Method)

Rajendran et al. verified the suppression of the absorbance of ABTS^●+^ in a concentration-dependent manner for artocarpin (**47**) and cycloartocarpin (**132**). The results demonstrate that the reaction with these compounds show small inhibitory effects even up to 4 min of reaction, when compared with the reaction of the positive control quercetin which is completed within 1 min. Artocarpin (**47)** has two hydroxyl groups in the B ring at 4′ and 6′and registered a TEAC value of 910 μM while cycloartocarpin (**132)** has a fused partially saturated six member heterocyclic ring between rings C and B and had a TEAC value of 690 μM. Meanwhile, the control quercetin possesses a catechol structure in the B ring, a C2=C3 double bond in conjunction a 3-OH and 4-carbonyl groups, allowing resonance stabilization for electron delocalization and therefore, an higher TEAC value (1230 μM) when compared with **47** and **132**. These results demonstrate the importance of electron delocalization across the molecule for stabilization of the aryloxyl radical [32].

The two prenylated flavones cycloartocarpesin B (**56**) and cudraflavone B (**65**) and three flavanones—euchrestaflavanone B (**94**), euchrestaflavanone C (**95**) and a novel flavanone A (**96**)—had similar ABTS^●+^ scavenging activity (IC_50_ 4.2–8.3 μM) to that of quercetin (IC_50_ 4.0 μM). In addition, the most active compound **65** had the same TEAC value (expresses the numbers of μmols of Trolox having an antioxidant capacity corresponding to 1.0 μmol of the test substance) than quercetin (TEAC value of 5.0). These results point out the importance of the prenyl group for the antioxidant effect against ABTS system [38].

Wu et al. synthesized three baicalein derivatives **73**–**75** possessing different prenylated chains at C-7 and evaluated their ABTS^●+^ scavenging activity. The order of potency was: parent baicalein (IC_50_ 5.5 ± 0.40 μM) > 7-prenylbaicalein (**73**, IC_50_ 8.8 ± 0.11 μM) ≈7-geranylbaicalein (**74**, IC_50_ 8.7 ± 0.28 μM) > 7-farnesylbaicalein (**75**, IC_50_ 10.6 ± 0.50 μM). Looking at the results, substitution of 7-hydroxyl group of baicalein by terpenoid groups led to a decrease in the scavenging activity and that the largest farnesyl group lead to the weakest scavenger. Even so, the scavenging activities of baicalein and its derivatives **73**–**75** were higher than that of Trolox (IC_50_ 12.6 ± 0.21 μM), the water-soluble vitamin E analogue with one hydroxyl group [43].

Among eight flavonoids isolated from *S. flavescens*, the chalcones kuraridin (**18**) and kuraridinol (**19**) were the most potent ABTS^●+^ scavengers with IC_50_ values of 10.45 ± 0.07 and 11.90 ± 2.77 μM, respectively. The remaining compounds (the flavonol kushenol C (**70**) and the flavanones leachianone (**89**), kushenol E (**90**), sophoraflavanone G (**91**), kurarinone (**92**) and kurarinol (**93**)) revealed IC_50_ values in the range of 14.08 to 28.84 μM, while the positive controls Trolox and L-ascorbic acid exhibited IC_50_ values of 24.57 ± 0.11 and 28.86 ± 0.02 μM, respectively. In addition, **70**, **18** and **19** possessed TEAC values of 1.88, 2.45, and 2.44, respectively, whereas those of the flavanones (**89**, **90**, **91**, **92** and **93**) were 1.28, 1.76, 1.05, 1.56, and 1.39, respectively [22].

Lan et al. isolated 14 flavonoids from *A. altilis*, but only five were evaluated in an ABTS^●+^ scavenging assay. Isocycloartobiloxanthone (**134**) showed a concentration-dependent scavenging behavior and was the most efficient scavenger, with an IC_50_ value of 7.2 ± 1.6 μM, similar to the positive control quercetin (IC_50_ 7.8 ± 2.1 μM). The order to potency for the other derivatives was: artogomezianone (**50**, IC_50_ 36.9 ± 2.3 μM) > 8-geranyl-3-(hydroxyprenyl)isoetin (**52**, IC_50_ 156.9 ± 5.3 μM) > artocarpin (**47**, IC_50_ 265.1 ± 4.3 μM). No activity was found for cyclocommunol (**131**, IC_50_ > 500 μM) [35].

Xanthohumol (**1**) had a maximum inhibition of ABTS^●+^ of 47% at 60 μM concentration and a TEAC value of 0.32 ± 0.09 μM [17]. In another study involving xanthohumol (**1**) and seven synthetic ester derivatives, 4-*O*-acetylxanthohumol (**25**, 160 μM TEAC/g) and 4-*O*-decanoylxanthohumol (**27**, 170 μM TEAC/g) displayed higher antioxidant effect than xanthohumol (**1**, 190 μM TEAC/g). The other tested compounds showed comparable or weaker ABTS scavenger properties than **1** (>200 μM TEAC/g) [27]. The ABTS radical scavenging profile of the chalcone desmethylxanthohumol (**3**) was expressed as the half-maximal effective concentration (EC_50_), presenting an EC_50_ of 0.54 ± 0.09 μM, slightly less than that of gallic acid (0.35 ± 0.01 μM) [29].

Isoxanthohumol (**21**) and 2′,4′,6′-trihydroxy-3′-prenylchalcone (**22**) displayed the highest TEAC values (4529.01 ± 2.44; 4170.66 ± 6.72) μM Trolox equivalents/g, respectively, among the flavonoids isolated from *H. teretifolium*. Isoglabranin (**99**), glabranin (**100**) and 7-methoxyisoglabranin (**101**) presented modest ABTS^●+^ scavenging effects and heliteretifolin (**20**) was completely inactive [24].

From the seven flavonoids isolated from *A. scortechinii* King it was only possible to determine the ABTS^●+^ scavenging potential of flemichapparin A (**15**, IC_50_ 199.7 μM), artonin E (**55**, IC_50_ 145.0 μM) and cycloartobiloxanthone (**140**, IC_50_ 269.0 μM). As in the DPPH^●^ assay, the positive control BHA used in this assay was a stronger scavenger than the analyzed compounds (IC_50_ 91.01 μM) [19].

Glyasperin A (**71** (IC_50_ 210.0 ± 2.7 μM) isolated from *M. pruinosa* leaf showed almost half the antioxidant potential than the positive control kaempferol (IC_50_ 111.0 ± 1.6 μM) in the ABTS^●+^ scavenging assay [41].

##### Ferric Reducing Antioxidant Power (FRAP) Method

A weak FRAP value was observed for the isoflavones alpinum isoflavone (**119**, 35.55 ± 1.23 μM Trolox equivalents), angustone C (**130**, 24.38 ± 1.15 μM Trolox equivalents) and 4′-acetylalpinum isoflavone (**129**, 2.32 ± 0.01 μM Trolox equivalents) isolated from *A. madreporica* when compared with the antioxidant quercetin (236.43 ± 6.2 μM Trolox equivalents) in the FRAP assay [53].

Xanthohumol (**1**) was a stronger scavenger in the ABTS^●+^ scavenging system than in the FRAP system, with 0.27 ± 0.04 μM Trolox equivalents in the FRAP assay system, and was completely inactive in the scavenging of DPPH^●^ [17].

Similarly to the ABTS assay, isoxanthohumol (**21**) and 2′,4′,6′-trihydroxy-3′-prenylchalcone (**22**) displayed the highest values (619.91 ± 1.97; 817.94 ± 4.26 μM ascorbic acid equivalents per mg dry weight, respectively), in the FRAP assay. Isoglabranin (**99**), glabranin (**100**) and 7-methoxyisoglabranin (**101**) presented weak FRAP effects and heliteretifolin (**20**) was completely inactive [24].

The FRAP value of chalcone dimer **44** was calculated from the calibration curve derived from dilutions of a vitamin C standard, measuring the decrease in absorption of the complex at 660 nm. Compound **44** exhibited better activity in the FRAP assay than the positive control, gallic acid (648.44 and 531.02 mg/mM, equivalent amounts of vitamin C) [29].

It was only possible to determine the FRAP potential of three of the seven flavonoids isolated from *A. scortechinii* King. Artocarpin (**47**), artonin E (**55**) and cycloartobiloxanthone (**140**) exhibited FRAP values of 0.19 ± 0.19, 1.32 ± 1.17 and 2.79 ± 0.19 Trolox equivalents, respectively. A closer look into the FRAP values of artocarpin (**47**) and artonin E (**55**) we may question the reliability of such results since the error associated is too high. The two positive controls used in this assay, BHA and HBT, provided values of 0.60 ± 0.06 and 1.89 ± 0.02 Trolox equivalents, respectively [19].

#### 4.1.2. Hydrogen Atom Transfer Mechanisms

##### Superoxide Radical Anion Scavenging Activity

Xanthohumol (**1**) was able to scavenge several reactive oxygen species, including the inhibition of superoxide anion radical (O_2_^•−^) production, generated by hypoxanthine/xanthine oxidase system and quantified by reduction of NBT, in a concentration-dependent manner, presenting a SC_50_ of 27.7 ± 4.9 μM. Furthermore, detection of uric acid proved that the result confirm the mechanism of action [14]. With the same detection technique, Lan et al. evaluated O_2_^•−^ scavenging potential of five flavonoids isolated from *A. altilis*. Artogomezianone (**50**) and artocarpin (**47**) exhibited moderate scavenging activities with IC_50_ values of 39.7 ± 3.3 and 94.1 ± 1.8 μM, respectively, when compared with the positive control quercetin (IC_50_ 3.9 ± 0.4 μM). 8-Geranyl-3-(hydroxyprenyl)isoetin (**52**), isocycloartobiloxanthone (**134**) and cyclocommunol (**131**) displayed weak inhibitory activities, with IC_50_ values higher than 300 μM [35].

In order to evaluate O_2_^•−^ scavenging activity, Ko et al. generated it using a xanthine/xanthine oxidase system and monitored the reduction of cytochrome c at 550 nm. The results indicated that none of the compounds isolated from *A. heterophyllus* Lam (cycloheterophyllin (**143**), artonin A (**144**) and artonin B (**145**)) inhibit xanthine oxidase activity or scavenge O_2_^•−^ [31]. A weak scavenging activity against O_2_^•−^ was registed by Fukai et al. for licochalcone A (**17**), morusin (**68**), licoricidin (**151**) and licorisoflavan A (**152**) [21]. A similar behavior was reported by Rajendran et al. for artocarpin (**47**) and cycloartocarpin (**132**), when compared to the positive controls, ascorbic acid and Trolox [32].

Four spectrophotometric detectors of O_2_^•−^ were used to study the reaction of xanthohumol (**1**) and 3-hydroxyxanthohumol (**32**) in the xanthine oxidase model: NBT at 560 nm, XTT at 510 nm, hydroxylamine at 540 nm, cytochrome c at 550 nm. Superoxide was also detected by formation of 2-hydroxyethidium formed from hydroethidine by xanthine/xanthine oxidase system and quantified after separation by HPLC. The results indicated that xanthohumol lacked superoxide scavenging activity in contrast to the 3′-hydroxy derivative, when sufficient concentration of NBT and other detectors such as XTT, hydroxylamine, cytochrome c and hydroethidine were used. In addition, xanthohumol (**1**) can moderately generate superoxide via auto-oxidation reaction [26].

Finally, Lin et al. evaluated the direct inhibition of xanthine oxidase enzymatic activity, by reacting the enzyme with the tested compounds and using xanthine as substrate. The reaction was monitored for 5 min at 295 nm. Xanthone-derivative artonol A (**142**, IC_50_ 43.3 ± 8.1 μM) was twice as active as an inhibitor of xanthine oxidase activity than the flavone cyclogeracommunin (**133**, IC_50_ 73.3 ± 19.1 μM), although considerably less active than the positive control allopurinol (IC_50_ 2.0 ± 0.7 μM) [34].

##### Oxygen Radical Absorbance Capacity (ORAC) Method

Ko et al. evaluated the ability of cycloheterophyllin (**143**), artonin A (**144**) and artonin B (**145**) to scavenge hydrophilic and lipophilic peroxyl (RO_2_^•^) radicals. Thus, **144** and **145** scavenged RO_2_^•^ generated by AAPH in aqueous media more efficiently than the positive control ascorbic acid. Due to the quenching fluorescent intensity of β-phycoerythrin by **143**, its effect in aqueous media was not evaluated. Additionally, none of the tested flavonoids scavenge RO_2_^•^ derived from 2,2′-azobis(2,4-dimethylvaleronitrile) in hexane [31].

Xantohumol (**1**) and isoxanthohumol (**102**) were tested as scavengers of both hydroxyl (HO^•^) and peroxyl (RO_2_^•^) radicals. Thus, β-phycoerythrin was used as a redox-sensitive fluorescent indicator protein, 2,2,-azobis-(2-amidinopropane) dihydrochloride as RO_2_^•^ generator and H_2_O_2_-CuSO_4_ system as HO^•^ generator. The results were expressed as ORAC units where 1 ORAC unit equals the net protection of β-phycoerythrin produced by 1 μM Trolox. Xanthohumol (**1**) was almost 9-fold and 3-fold more active than Trolox, in scavenging HO^•^ and RO_2_^•^, respectively, at 1 μM concentration. Isoxanthohumol (**102**) presented a similar effect in scavenging HO^•^ as Trolox and was even more active than xanthohumol (**1**) in RO_2_^•^scavenging, especially at 5 μM concentration [14]. Using the same experimental procedure, heliteretifolin (**20**), isoxanthohumol (**21**), 2′,4′,6′-trihydroxy-3′-prenylchalcone (**22**), isoglabranin (**99**), glabranin (**100**) and 7-methoxyisoglabranin (**101**) isolated from *H. terefolium* exhibited a weak scavenging activity in ORAC assay for both HO^•^ and RO_2_^•^ [24].

Fluorescein can be used as generator of RO_2_^•^ by the application of 2,2′-azobis(2-methyl-propionamide) dihydrochloride as a free radical initiator. Vogel et al. tested eleven chalcones (xanthohumol (**1**), xanthogalenol (**2**), desmethylxanthohumol (**3**), 4′-*O*-methylxanthohumol (**24**), 3-hydroxyxanthohumol (**32**), 2,2′,4′-trihydroxy-6′-methoxy-3′-prenylchalcone (**34**), 2′,3,4′-trihydroxy-6′-methoxy-3′-prenylchalcone (**35**), 2′,3,4′,5-tetrahydroxy-6′-methoxy-3′-prenylchalcone (**36**), 2′,3,4,4′,5-pentahydroxy-6′-methoxy-3′-prenylchalcone (**37**), 2′,4′-dihydroxy-3,4,6′-trimethoxy-3′-prenylchalcone (**38**), and 4,6′-dihydroxy-2,′4′-dimethoxy-3′-prenylchalcone (**39**)) that revealed potent antioxidant activity from 0.9 to 3.8 Trolox equivalents in a concentration range between 0.1 and 2.0 μM. The most active compounds were **3** and **24** with 3.8 ± 0.5 and 3.8 ± 0.4 Trolox equivalents, respectively [25]. This research group also tested other chalcones (xanthohumol (**1**), 5′-prenyl-xanthohumol (**5**), xanthohumol H (**8**), xanthohumol C (**9**), 1″,2″-dihydroxanthohumol C (**10**), 3′-geranylchalconaringenin (**11**), 3′-geranyl-6′-*O*-methylchalconaringenin (**12**) and 2′-*O*-methyl-3′-prenylchalconaringenin (**13**)) and the ORAC-fluorescein assay revealed a high activity from 1.7 to 5.2 Trolox equivalents in a concentration range between 0.1 and 1.0 μM. Chalcones **8** and **13** were the most potent scavengers, with 4.8 and 5.2 Trolox equivalents, respectively [16]. Next, additional seven chalcones (4-*O*-methylxanthohumol (**24**), 4-*O*-acetylxanthohumol (**25**), 3-methoxyxanthohumol (**33**), 3-hydroxyxanthohumol C (**40**), 3-methoxyxanthohumol C (**41**), 3-hydroxyxanthohumol H (**42**) and 3-methoxyxanthohumol H (**43**)) were evaluated in a concentration range of 0.25 to1.5 μM and presented ORAC values between 0.6 and 3.9 Trolox equivalents. The most active compounds were **42** and **43** showing 3.9 ± 0.5 and 3.0 ± 0.2 Trolox equivalents, respectively, while **24** and **25** were the less active ones (< 2 Trolox equivalents). The remaining chalcones showed moderate scavenging activity with 2.0 and 2.6 Trolox equivalents [28].

Like in the other radical scavenging assays (DPPH^•^ and ABTS^•+^), glyasperin A (**71**, 2.5 ± 0.6 μmol Trolox equivalents/mg) isolated from *M. pruinosa* showed a weaker RO_2_^•^ scavenging potential than the positive control kaempferol (34.9 ± 0.6 μmol Trolox equivalents/mg) [41].

##### Hydroxyl Radical Detected by ESR

The ability of the prenylated flavonoids to scavenge HO^•^ in a hydrophilic environment can also be measured by ESR spectroscopy, where HO^•^ radicals generated by a Fenton-type reaction are trapped as DMPO spin adducts, giving rise to the corresponding ESR signals. The intensity of the DMPO-OH spin adduct signal is reduced in the presence of radical scavengers and the results are given as the difference of the respective ESR signal intensities of the sample with and without the flavonoid. Lee et al. isolated five flavonoids from *C. tricuspidata* (cycloartocarpesin B (**56**), cudraflavone B (**65**), euchrestaflavanone B (**94**), euchrestaflavanone C (**95**) and novel flavanone A (**96**)) and evaluated their ability to scavenge HO^•^. Compounds **94**, **56** and **65** were more active (IC_50_ values of 34.6 ± 2.7 μM, 46.9 ± 6.5 μM and 40.6 ± 2.1 μM, respectively) than Trolox, with an IC_50_ value of 48.2 ± 2.3 μM. Euchrestaflavanone C (**95**) and novel flavanone A (**96**) were moderate scavengers with IC_50_ values 52.5 ± 3.6 μM and 75.3 ± 4.8 μM, respectively [38].

##### Other ROS/RNS Scavenging Activity

Hydrogen peroxide (H_2_O_2_) scavenging capacity can also be related to the antioxidant activity of a matrix. This ROS is generated in vivo, under physiological conditions, by peroxisomes, by several oxidative enzymes and by dismutation of superoxide radical, catalyzed by superoxide dismutase. Ko et al. evaluated the content of hydrogen peroxide indirectly using a catalase-based method. The reaction mixture containing H_2_O_2_ and test compound in phosphate buffer pH 7.4 was incubated at 25 °C for 40 min. Then, catalase was added and O_2_ release was monitored polarographically for 0.8 min. The amount of H_2_O_2_ remaining was calculated using a standard curve made of O_2_ production vs. H_2_O_2_ concentration (0.1–2.0 μM). At 100 μM concentration, cycloheterophyllin (**143**), artonin A (**144**) and artonin B (**145**) were not able to react directly with H_2_O_2_ since no significant loss of H_2_O_2_ was observed [31].

The ONOO^−^ scavenging activity reported by Jung et al. was measured by monitoring the ONOO^−^ induced oxidation of non-fluorescent DHR to fluorescent rhodamine 123. The assay was performed at 37 °C, reacting the tested compounds dissolved in 10% DMSO, ONOO^−^ and DHR 123 and the fluorimetric signal detected after a 5 min incubation period. The fluorescence intensity of the oxidized DHR 123 was measured at the excitation and emission wavelengths of 485 nm and 530 nm, respectively, and the results were expressed the percent inhibition of oxidation of DHR 123. L-Penicillamine was used as the positive control. From the eight flavonoids analyzed (the chalcones kuraridin (**18**) and kuraridinol (**19**), the flavonol kushenol C (**70**) and the flavanones leachianone (**89**), kushenol E (**90**), sophoraflavanone G (**91**), kurarinone (**92**) and kurarinol (**93**)), the flavonol **70** (IC_50_ 0.62 ± 0.01 μM) was the most active scavenger, even better than the positive control, L-penicillamine (IC_50_ 2.37 ± 0.09 μM). The chalcones **18** and **19** exhibited IC_50_ values of 2.89 and 2.19 μM, respectively, while flavanones **89**–**93** were less active ones, with IC_50_ values of 3.17, 3.35, 7.30, 3.42, and 3.64 μM, respectively [22]. As mentioned before for the DPPH^●^ scavenging activity, the structural features of flavonols may enhance also the ONOO^−^ scavenging potential, when compared to flavanone or chalcone derivatives.

#### 4.1.3. Metal Chelation

Ko et al. reported that cycloheterophyllin (**143**), artonin A (**144**) and artonin B (**145**) had peaks at 310 and 418, 302 and 380, and 295 and 400 nm, respectively. No changes in the absorbance or spectral shift were observed after the addition of Fe^2+^, Fe^3+^ or Cu^2+^ solutions to the reaction mixtures containing **143**, **144** and **145** [31]. A series of other flavonoids were tested by Miranda et al.: xanthohumol (**1**), xanthogalenol (**2**), desmethylxanthohumol (**3**), isoxanthohumol (**102**) and 8-prenylnaringenin (**113**). Spectra (200–600 nm) were recorded after preparation of the mixtures and 10 min later, in the absence and in presence of copper ions. Small variations were observed in the spectra of **1**, **102** and **113**. The chalcones **2** and **3** developed new maxima around 290 nm over a 10-min period, which was attributed to conversion of the chalcones to their isomeric flavanones rather than chelation of copper ions. The importance of 3′,4′-dihydroxy substituents (catechol moieties) on the B-ring for copper or iron chelate formation is known and none of the flavonoids is this study had such a profile [12]. In continuation of their studies, **1**, **2** and **3** were also tested for their chelation of iron ions, recording the absorbance between 190 and 600 nm, and no changes were induced by this metal in the UV-vis spectra [13]. Unlike Miranda’s work, Dufall et al. tested three prenylated flavonoids bearing a catechol unit in the B-ring: 6,8-diprenyleriodictyol (**97**), dorsmanin C (**67**) and dorsmanin F (**98**). Methanolic solutions of the compounds were mixed with copper solution and their interaction was measured between 200 and 800 nm after 10 s and compared with the flavonoid alone. Interestingly, only **67** showed any interaction with Cu^2+^ ions indicated by significant bathochromic shift in major absorbance bands upon addition of equimolar concentrations of Cu^2+^ ions. These results led us to infer t the importance of the catechol unit for the chelating properties but also of the presence of C2=C3 double bond in combination with a 3-OH, the structural characteristics of flavonol **67** absent in flavanones **97** and **98** [37]. Meanwhile, 3,5,7,2′-tetrahydroxy-6-methoxy-8-prenylflavanone, known as floranol (**76**) exhibits two absorption bands at 297 and 340 nm related to the π-π* transitions of chromophores A and B, respectively, which were significantly changed by both coordination to Cu^2+^ and Fe^3+^ ions. The authors pointed out that these metals probably bind to a site on A-ring through a bidentate coordination involving the 5-OH and 4-carbonyl groups [44].

### 4.2. In Vivo Methods

#### 4.2.1. Lipid Peroxidation

Ko et al. studied lipid peroxidation promoted by iron ions in rat brain homogenate and measured by the decrease of absorbance at 532 nm. Tetramethoxypropane was used as a standard, and the results were expressed as nanomoles of MDA equivalents per milligram of protein of rat brain homogenates. Cycloheterophyllin (**143**), artonin A (**144**) and artonin B (**145**) inhibited Fe(II)-induced lipid peroxidation in a concentration-dependent manner with IC_50_ values calculated to be 0.96 ± 0.21, 0.47 ± 0.24 and 0.71 ± 0.13 μM, respectively. BHT also inhibited this Fe(II)-induced lipid peroxidation with an IC_50_ 1.33 ± 0.05 μM. In contrast, no effect on Fe(II)-induced lipid peroxidation in rat brain homogenate was observed for artocarpin (**47**) and artocarpetin A (**58**) at 100 μM concentration [31].

Low inhibitory activity on Fe(II)-induced microsomal lipid peroxidation was observed for the flavonoids isolated from *H. teretifolium* heliteretifolin (**20**), isoxanthohumol (**21**), 2′,4′,6′-trihydroxy-3′-prenylchalcone (**22**), isoglabranin (**99**), glabranin (**100**) and 7-methoxyisoglabranin (**101**), with IC_50_ values superior to 21 μg/mL, when compared with the positive control, epigallocatechin gallate (IC_50_ 0.929 μg/mL) [24].

Rodriguez et al. evaluated the inhibition of metal-ion iron-dependent and independent (*tert*-butyl hydroperoxide, THB) lipid peroxidation in rat liver microsomes by a series of nine flavonoids (xanthonhumol (**1**), xanthogalenol (**2**), desmethylxanthohumol (**3**), 4′-*O*-methylxanthohumol (**4**) 5′-prenylxanthohumol (**5**), dehydrocycloxanthohumol (**6**), dehydrocycloxanthohumol hydrate (**7**) 3′-geranylchalconaringenin (**11**) and isoxanthohumol (**102**)). For the iron-dependent assays, two different systems were applied to induce lipid peroxidation, FeSO_4_/ascorbic acid and FeCl_3_/ADP/NADPH. The results on the lipid peroxidation induced by iron/ascorbate pointed out that all compounds respond in a concentration-dependent manner. Prenylated derivatives were good inhibitors at 5 and 25 μM concentration and generally more effective than the non-prenylated derivatives. Xanthohumol (**1**), desmethylxanthohumol (**3**) and 5′-prenylxanthohumol (**5**) exhibited IC_50_ values of 6.0, 6.0 and 5.0 μM, respectively. In the Fe^3+^-ADP/NADPH-induced lipid peroxidation assay at 5 μM concentration, only **1**, **3**, **5** and **24** were efficient inhibitors; meanwhile at 25 μM concentration all nine prenylated flavonoids were effective in preventing lipid peroxidation, being **1**, **3** and **5** the most active ones. All prenylated flavonoids were able to prevent lipid peroxidation induced by THP at 5 and 25 μM concentration. For the highest concentration, the best inhibitors were **3**, **5** and **11** [13].

Studies on artocarpin (**47**) and cycloartocarpin (**132**) as inhibitors of lipid peroxidation was studied by following Mb(IV) reduction, induced by fatty acid, arachidonic acid. Decrease in the absorbance at 532 nm was recorded and the results were expressed as % inhibition of TBARS. At 100 μM concentration, cycloartocarpin (**132**, 34% inhibition) inhibited the formation of MDA more efficiently than ascorbic acid (27% inhibition) and even better than artocarpin (**47**, 24% inhibition) [32].

The effects of nine prenylated flavonoids (propolin A (**103**), propolin B (**104**), propolin E (**105**), prokinawan (**106**), nymphaeol A (**107**) nymphaeol B (**108**), nymphaeol C (**109**), isonymphaeol B (**110**) and 3′-geranylnaringenin (**111**)) on the oxidation of β-carotene/linoleic acid emulsion was monitored spectrophotometrically by measuring the absorbance at 470 nm over a 60 min period of incubation and the results expressed as IC_50_ values. Compounds **103**, **104**, **108**, **109** and **110** (possessing a geranyl group on their B-ring) showed higher activity (IC_50_ values ranging from 5.8 to 12.7 μM) than compounds **106** and **107** (possessing a geranyl group on their A-ring, with IC_50_ values of 35.8 and 28.9 μM, respectively), suggesting the importance of the position of the geranyl group in the flavonoid skeleton for the antioxidant activity. In addition, the absence of the catechol unit decreased drastically the antioxidant potential, as observed for derivative **105** (IC_50_ 73.7 ± 2.3 μM) and **110** (IC_50_ 98.3 ± 3.3 μM) [47].

#### 4.2.2. LDL Oxidation

Cycloheterophyllin (**143**), artonin A (**144**) and artonin B (**145**) inhibited Cu(II)-catalyzed oxidation of human LDL, as measured by fluorescence intensity, TBARS formation, conjugated diene formation and electrophoretic mobility in a concentration-dependent manner. Specifically, Cu-induced oxidation of LDL for 12 h led to an increased content of TBARS (212.3 ± 21.4 nmol/mg protein) when compared with TBARS level in unstimulated LDL (1.4 ± 0.5 nmol/mg protein), however, in the presence of **143**, **144** and B **145** at 30 μM concentration, the levels of TBARS formation were drastically reduced (2.5 ± 0.8, 6.0 ± 1.5 and 4.5 ± 1.2 nmol/mg protein, respectively) [31].

After 5 h of incubation, Cu(II)-induced oxidation of LDL was monitored by the formation of conjugated dienes and TBARS and the loss of tryptophan fluorescence by a group of 16 prenylated flavonoids (xanthohumol (**1**), xanthogalenol (**2**), desmethylxanthohumol (**3**), 4′-*O*-methyl-xanthohumol (**4**), 5′-prenylxanthohumol (**5**), dehydrocycloxanthohumol (**6**), dehydrocyclo-xanthohumol hydrate (**7**) 3′-geranylchalconaringenin (**11**), 4′-*O*-5′-*C*-diprenylxanthohumol (**23**), tetrahydroxanthohumol (**45**), isoxanthohumol (**102**), 6-prenylnaringenin (**112**), 8-prenylnaringenin (**113**), 6,8-diprenylnaringenin (**114**), 6-geranylnaringenin (**115**) and 8-geranylnaringenin (**116**)). At concentrations of 5 μM, all the prenylchalcones tested inhibited in some extension the formation of conjugated dienes induced by copper sulfate, while for 25 μM concentration complete inhibition of conjugated dienes formation occurred. The prenylchalcones showed higher inhibitory effects in the formation of conjugate dienes and on TBARS formation in LDL than the prenylflavanones, both at 5 and 25 μM concentration. Interestingly, at 12.5 μM concentration, **1** showed higher inhibition of LDL oxidation than α-tocopherol, but lower than the flavonol quercetin. Concerning the reduction in tryptophan fluorescence induced by copper, chalcones **1**, **3** and **11** inhibited in higher extension tryptophan oxidation in contrast to flavanones **113** and **102** [12].

LDL oxidation was induced by the peroxynitrite generator, 3-morpholinosydnonimine (SIN-1), and measured by the formation of conjugated dienes and TBARS. Conjugated diene formation was monitored by recording the absorbance at 250 nm every 30 min for 8 h and then at 18 and 24 h. After a 24 h incubation at 25 °C, TBARS were measured. The results pointed out that xanthohumol (**1**) inhibited SIN-1-induced oxidation of LDL in a dose-dependent manner in the assay for conjugated diene formation and in the TBARS assay. High inhibitions were also recorded in the TBARS formation for a series of other prenylated chalcones at 5 μM, (xanthogalenol (**2**), desmethylxanthohumol (**3**), 5′-prenylxanthohumol (**5**), dehydrocycloxanthohumol (**6**) and dehydro- cycloxanthohumol hydrate (**7**)), while the highly lipophilic prenylchalcones 3′-geranyl-chalconaringenin (**11)** and 4′-*O*-5′-*C*-diprenylxanthohumol (**23**) as well as prenylated flavanones **102** and **112–116** were considerably less active. Generally, chalcones are more potent inhibitors of LDL oxidation than flavanones due to their α,β -unsaturated keto group that can act as a Michael acceptor system for peroxynitrite. The introduction of additional prenyl groups further enhances the nucleophilicity of the 2′-OH group but also increases the compound’s lipophilicity and reduces its water solubility, making their oxidation products less effective ROS/RNS scavengers in aqueous medium [15].

The prenylated flavonoids 6,8-diprenyleriodictyol (**97**), dorsmanin C (**67**) and dorsmanin F (**98**) were effective inhibitors, in a concentration-dependent manner, of Cu(II)-mediated oxidation of LDL with IC_50_ values < 1 μM. In this assay, lipid hydroperoxides were measured by the ferrous oxidation-xylenol orange assay and the results expressed as inhibition of lipid hydroperoxide formation. 6,8-Diprenyleriodictyol (**97**) exhibited similar inhibitory potential to the positive control quercetin [37].

Five derivatives (cycloartocarpesin B (**56**), cudraflavone B (**65**), euchrestaflavanone B (**94**), euchrestaflavanone C (**95**) and novel flavanone A (**96**)) isolated from *C. tricuspidata* were assessed for their potential as inhibitors of Cu(II)-induced oxidation of LDL. All compounds exhibited modest antioxidant activity against LDL oxidation in TBARS assay with IC_50_ values ranging from 27.2 to 65.6 μM, in comparison with an IC_50_ of 3.6 μM obtained for the positive control probucol [39].

The antioxidant activity of floranol (**76**) isolated from the roots of *Dioclea grandiflora*, was evaluated by the inhibition of Cu(II)-induced oxidation of human LDL by measuring the formation of conjugated dienes. Floranol (**76**) inhibited the LDL oxidation, in a dose-dependent manner. Thus, in the absence of **76** a lag phase of 33 ± 1 min was measured; while in the presence of **76**, lag-phases of 68 ± 1 and 178 ± 2 min were obtained for 3 and 10 μM concentration, respectively. Concentrations above 30 μM practically prevent LDL oxidation [44].

## 5. Conclusions

A good number of prenylated flavonoids (more than 150 derivatives) have been studied over the past two decades focusing on their antioxidant properties. Most of them were isolated from different parts of plants belonging to the Moraceae and Fabaceae families, with a limited number of derivatives from the Apiaceae, Asteraceae, Cannabaceae, and Euphorbiaceae. Few *O*- and *C*-derivatives obtained by synthesis belong to the chalcone, flavone and flavanone class of compounds. A detailed description of all in vitro and in vivo methodologies used to study the antioxidant effects of natural and synthetic derivatives were made, including reactants, temperature, time and type of detection. DPPH radical scavenging assay is undoubtedly the most frequently used technique, probably due to its simplicity and low cost. From the published results, most of the analyzed prenylated flavonoids exhibited high inhibitory effects and some structure-activity relationships were also described. However, it is not easy to make a comparison between compounds since different methods and positive controls were applied. In conclusion, the importance of prenyl groups for the antioxidant properties of flavonoids in several in vitro and in vivo models is highlighted in this review.

## Figures and Tables

**Table 1:** Naturally occurring prenylated chalcones with antioxidant activity.

N	Trivial Name	Species	Extract	Ref.
		Cannabaceae		
**1**	xanthohumol	*Humulus lupulus*	hop	[12,13,14,15,16,17]
**2**	xanthogalenol	*Humulus lupulus*	hop	[12,14]
**3**	desmethylxanthohumol	*Humulus lupulus*	hop	[12,13,14]
**4**	4′-*O*-methylxanthohumol	*Humulus lupulus*	hop	[12,13,14]
**5**	5′-prenylxanthohumol	*Humulus lupulus*	hop	[12,13,14,16]
**6**	dehydrocycloxanthohumol	*Humulus lupulus*	hop	[12,13,14]
**7**	dehydrocycloxanthohumol hydrate	*Humulus lupulus*	hop	[12,13,14]
**8**	xanthohumol H	*Humulus lupulus*	hop	[16]
**9**	xanthohumol C	*Humulus lupulus*	hop	[16]
**10**	1″,2″-dihydroxanthohumol C	*Humulus lupulus*	hop	[16]
**11**	3′-geranylchalconaringenin	*Humulus lupulus*	hop	[12,13,14,16]
**12**	3′-geranyl-6′-*O*-methylchalconaringenin	*Humulus lupulus*	hop	[16]
**13**	2′-*O*-methyl-3′-prenylchalconaringenin	*Humulus lupulus*	hop	[16]
		Moraceae		
**14**	isobavachalcone	*Artocarpus anisophyllus*	heartwood and leaf	[18]
**15**	flemichapparin A	*Artocarpus scortechinii*	leaf and stem bark	[19]
**14**	isobavachalcone	*Dorstenia barteri*	twig and leaf	[20]
**16**	bartericin A	*Dorstenia barteri*	twig and leaf	[20]
		Fabaceae		
**17**	licochalcone A	*Glycyrrhiza inflate*		[21]
**18**	kuraridin	*Sophora flavescens*	root	[22,23]
**19**	kuraridinol	*Sophora flavescens*	root	[22,23]
		Asteraceae		
**20**	heliteretifolin	*Helichrysum teretifolium*	aerial part	[24]
**21**	isoxanthohumol	*Helichrysum teretifolium*	aerial part	[24]
**22**	2′,4′,6′-trihydroxy-3′-prenylchalcone	*Helichrysum teretifolium*	aerial part	[24]
		Obtained by synthesis		
**1**	xanthohumol	synthesis		[16,25,26,27]
**2**	xanthogalenol	synthesis		[25]
**3**	desmethylxanthohumol	synthesis		[25]
**4**	4′-*O*-methylxanthohumol	synthesis		[25]
**23**	4′-*O*-5′-*C*-diprenylxanthohumol	synthesis		[12,13,14]
**24**	4-*O*-methylxanthohumol	synthesis		[28]
**25**	4-*O*-acetylxanthohumol	synthesis		[27,28]
**26**	4,4′-di-*O*-acetylxanthohumol	synthesis		[27]
**27**	4-*O*-decanoylxanthohumol	synthesis		[27]
**28**	4-*O*-dodecanoylxanthohumol	synthesis		[27]
**29**	4,4′-di-*O*-dodecanoylxanthohumol	synthesis		[27]
**30**	4-*O*-pivaloylxanthohumol	synthesis		[27]
**31**	4,4′-di-*O*-pivaloylxanthohumol	synthesis		[27]
**32**	3-hydroxyxanthohumol	synthesis		[25,26]
**33**	3-methoxyxanthohumol	synthesis		[28]
**34**	2,2′,4′-trihydroxy-6′-methoxy-3′-prenylchalcone	synthesis		[25]
**35**	2′,3,4′-trihydroxy-6′-methoxy-3′-prenylchalcone	synthesis		[25]
**36**	2′,3,4′,5-tetrahydroxy-6′-methoxy-3′-prenylchalcone	synthesis		[25]
**37**	2′,3,4,4′,5-pentahydroxy-6′-methoxy-3′-prenylchalcone	synthesis		[25]
**38**	2′,4′-dihydroxy-3,4,6′-trimethoxy-3′-prenylchalcone	synthesis		[25]
**39**	4,6′-dihydroxy-2,′4′-dimethoxy-3′-prenylchalcone	synthesis		[25]
**40**	3-hydroxyxanthohumol C	synthesis		[28]
**41**	3-methoxyxanthohumol C	synthesis		[28]
**42**	3-hydroxyxanthohumol H	synthesis		[28]
**43**	3-methoxyxanthohumol H	synthesis		[28]
**44**	(2*E*,2′*E*)-1,1′-[methylenebis(5,7-dihydroxy-2,2-dimethyl-2*H*-chromene-6,8-diyl)]bis[3-(2,3,4-trihydroxyphenyl)prop-2-en-1-one]	synthesis		[29]

**Table 2:** Chemical structures of 3′-prenylated chalcones **1**–**5**, **13**, **14** and **22**–**39** with antioxidant activity.

N	Comp.	R^2^	R^3^	R^4^	R^5^	R^2′^	R^4′^	R^5′^	R^6′^
		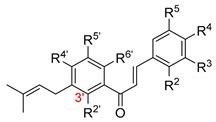							
**1**	xanthohumol	H	H	OH	H	OH	OH	H	OMe
**2**	xanthogalenol	H	H	OH	H	OH	OMe	H	OH
**3**	desmethylxanthohumol	H	H	OH	H	OH	OH	H	OH
**4**	4′-*O*-methylxanthohumol	H	H	OH	H	OH	OMe	H	OMe
**5**	5′-prenylxanthohumol	H	H	OH	H	OH	OH	prenyl	OMe
**13**	2′-O-methyl-3′-prenylchalconaringenin	H	H	OH	H	OMe	OH	H	OH
**14**	isobavachalcone	H	H	OH	H	OH	OMe	H	H
**21**	isoxanthohumol	H	H	H	H	OH	OMe	H	OH
**22**	2′,4′,6′-trihydroxy-3′-prenylchalcone	H	H	H	H	OH	OH	H	OH
**23**	4′-*O*-5′-*C*-diprenylxanthohumol	H	H	OH	H	OH	Oprenyl	prenyl	OMe
**24**	4-*O*-methylxanthohumol	H	H	OMe	H	OH	OH	H	OMe
**25**	4-*O*-acetylxanthohumol	H	H	OAc	H	OH	OH	H	OMe
**26**	4,4′-di-*O*-acetylxanthohumol	H	H	OAc	H	OH	OAc	H	OMe
**27**	4-*O*-decanoylxanthohumol	H	H	Odecanoyl	H	OH	OH	H	OMe
**28**	4-*O*-dodecanoylxanthohumol	H	H	Ododecanoyl	H	OH	OH	H	OMe
**29**	4,4′-di-*O*-dodecanoylxanthohumol	H	H	Ododecanoyl	H	OH	Ododecanoyl	H	OMe
**30**	4-*O*-pivaloylxanthohumol	H	H	Opivaloyl	H	OH	OH	H	OMe
**31**	4,4′-di-*O*-pivaloylxanthohumol	H	H	Opivaloyl	H	OH	Opivaloyl	H	OMe
**32**	3-hydroxyxanthohumol	H	OH	OH	H	OH	OH	H	OMe
**33**	3-methoxyxanthohumol	H	OMe	OH	H	OH	OH	H	OMe
**34**	2,2′,4′-trihydroxy-6′-methoxy-3′-prenylchalcone	OH	H	H	H	OH	OH	H	OMe
**35**	2′,3,4′-trihydroxy-6′-methoxy-3′-prenylchalcone	H	OH	H	H	OH	OH	H	OMe
**36**	2′,3,4′,5-tetrahydroxy-6′-methoxy-3′-prenylchalcone	H	OH	H	OH	OH	OH	H	OMe
**37**	2′,3,4,4′,5-pentahydroxy-6′-methoxy-3′-prenylchalcone	H	OH	OH	OH	OH	OH	H	OMe
**38**	2′,4′-dihydroxy-3,4,6′-trimethoxy-3′-prenylchalcone	H	OMe	OMe	H	OH	OH	H	OMe
**39**	4,6′-dihydroxy-2,′4′-dimethoxy-3′-prenylchalcone	H	H	OH	H	OMe	OMe	H	OH

**Table 3:** Natural occurrence of prenylated flavones with antioxidant activity.

N	Trivial Name	Species	Extract	Ref.
		Moraceae		
**47**	artocarpin	*Artocarpus altilis*	heartwood and cortex	[35]
**48**	artoflavone A	*Artocarpus altilis*	heartwood and cortex	[35]
**49**	hydroxyartoflavone A	*Artocarpus altilis*	heartwood and cortex	[35]
**50**	artogomezianone	*Artocarpus altilis*	heartwood and cortex	[35]
**51**	10-oxoartogomezianone	*Artocarpus altilis*	heartwood and cortex	[35]
**52**	8-geranyl-3-(hydroxyprenyl)isoetin	*Artocarpus altilis*	heartwood and cortex	[35]
**53**	8-geranylapigenin	*Artocarpus altilis*	heartwood and cortex	[35]
**47**	artocarpin	*Artocarpus anisophyllus*	heartwood and leaf	[18]
**54**	4′,5-dihydroxy-6,7-(2,2-dimethylpyrano)-2′-methoxy-8-(γ,γ-dimethyl)allylflavone	*Artocarpus anisophyllus*	heartwood and leaf	[18]
**55**	artonin E	*Artocarpus communis*	plant	[21]
**48**	artoflavone A	*Artocarpus communis*	cortex of root	[34]
**56**	cycloartocarpesin B	*Artocarpus elasticus*	leaf	[30]
**57**	artelastoheterol	*Artocarpus elasticus*	root bark	[34]
**47**	artocarpin	*Artocarpus heterophyllus*	plant	[31]
**58**	artocarpetin A	*Artocarpus heterophyllus*	plant	[31]
**49**	artocarpin	*Artocarpus incisa*	heartwood	[32]
**47**	artocarpin	*Artocarpus integer*	heartwood	[36]
**59**	cudraflavone C	*Artocarpus integer*	heartwood	[36]
**55**	artonin E	*Artocarpus nobilis*	root bark	[33]
**60**	2′-*O*-methylartonin E	*Artocarpus nobilis*	root bark	[33]
**61**	2′-*O*-methylisoartonin E	*Artocarpus nobilis*	root bark	[33]
**62**	2′-*O*-methyldihydroisoartonin E	*Artocarpus nobilis*	root bark	[33]
**63**	2′-*O*-methylartonin V	*Artocarpus nobilis*	root bark	[33]
**47**	artocarpin	*Artocarpus scortechinii*	leaf and stem bark	[19]
**55**	artonin E	*Artocarpus scortechinii*	leaf and stem bark	[19]
**54**	4′,5-dihydroxy-6,7-(2,2-dimethylpyrano)-2′-methoxy-8-(γ,γ-dimethyl)allyflavone	*Artocarpus scortechinii*	leaf and stem bark	[19]
**64**	macakurzin C	*Artocarpus scortechinii*	leaf and stem bark	[19]
**56**	cycloartocarpesin B	*Cudrania tricuspidata*	root bark	[38,39]
**65**	cudraflavone B	*Cudrania tricuspidata*	root bark	[38,39]
**66**	6-prenylapigenin	*Dorstenia kameruniana*	twig and leaf	[20]
**67**	dorsmanin C	*Dorstenia mannii*	twig and leaf	[20]
**68**	morusin	*Morus alba*	root bark	[21]
		Fabaceae		
**69**	3,5,2′,4′-tetrahydroxy-6″,6″-dimethylpyrano-(2″,3″:7,6)-8-prenylflavone	*Eriosema chinense*	root	[40]
**70**	kushenol C	*Sophora flavescens*	root	[22]
		Euphorbeaceae		
**71**	glyasperin A	*Macaranga gigantea*	leaf	[42]
**72**	broussoflavonol F	*Macaranga gigantea*	leaf	[42]
**71**	glyasperin A	*Macaranga pruinosa*	leaf	[41]
		Obtained by synthesis		
**73**	7-*O*-prenylbaicalein	synthesis		[16,43]
**74**	7-*O*-geranylbaicalein	synthesis		[43]
**75**	7-*O*-farnesylbaicalein	synthesis		[43]

**Table 4:** Natural occurrence of prenylated flavanones with antioxidant activity.

N	Trivial Name	Species	Extract	Ref.
		Fabaceae		
**76**	floranol	*Dioclea grandiflora*	root	[44]
**77**	khonklonginol A	*Eriosema chinense*	root	[40]
**78**	2‴,3‴-epoxykhonklonginol A	*Eriosema chinense*	root	[40]
**79**	lupinifolinol	*Eriosema chinense*	root	[40]
**80**	3-*epi*-lupinifolinol	*Eriosema chinense*	root	[40]
**81**	2-hydroxylupinifolinol	*Eriosema chinense*	root	[40]
**82**	flemichin D	*Eriosema chinense*	root	[40]
**83**	4′-*O*-methyllicoflavanone	*Erythrina orientalis*	stem bark	[46]
**84**	5,7-dihydroxy-6-methyl-8-prenylflavanone	*Eysenhardtia platycarpa*	leaf	[45]
**85**	5,7-dihydroxy-4′-methoxy-6-methyl-8-prenylflavanone	*Eysenhardtia platycarpa*	leaf	[45]
**86**	5,7-dihydroxy-6-prenylflavanone	*Eysenhardtia platycarpa*	leaf	[45]
**87**	5-hydroxy-7-methoxy-6-prenylflavanone	*Eysenhardtia platycarpa*	leaf	[45]
**88**	5,7-dihydroxy-4′-methoxy-8-prenylflavanone	*Eysenhardtia platycarpa*	leaf	[45]
**89**	leachianone	*Sophora flavescens*	root	[22]
**90**	kushenol E	*Sophora flavescens*	root	[22]
**91**	sophoraflavanone G	*Sophora flavescens*	root	[22]
**92**	kurarinone	*Sophora flavescens*	root	[22]
**93**	kurarinol	*Sophora flavescens*	root	[22]
		Moraceae		
**88**	5,7-dihydroxy-4′-methoxy-8-prenylflavanone	*Artocarpus anisophyllus*	heartwood and leaf	[18]
**94**	euchrestaflavanone B	*Cudrania tricuspidata*	root bark	[38,39]
**95**	euchrestaflavanone C	*Cudrania tricuspidata*	root bark	[38,39]
**96**	novel flavanone A	*Cudrania tricuspidata*	root bark	[38,39]
**97**	6,8-diprenyleriodictyol	*Dorstenia mannii*	twig and leaf	[20,37]
**98**	dorsmanin F	*Dorstenia mannii*	twig and leaf	[20,37]
		Asteraceae		
**99**	isoglabranin	*Helichrysum teretifolium*	aerial part	[24]
**100**	glabranin	*Helichrysum teretifolium*	aerial part	[24]
**101**	7-methoxyisoglabranin	*Helichrysum teretifolium*	aerial part	[24]
		Cannabaceae		
**102**	isoxanthohumol	*Humulus lupulus*	hop	[12,13,14]
		Obtained from propolis		
**103**	propolin A	Japanese	ethanol	[47]
**104**	propolin B	Japanese	ethanol	[47]
**105**	propolin E	Japanese	ethanol	[47]
**106**	prokinawan	Japanese	ethanol	[47]
**107**	nymphaeol A	Japanese	ethanol	[47]
**108**	nymphaeol B	Japanese	ethanol	[47]
**109**	nymphaeol C	Japanese	ethanol	[47]
**110**	isonymphaeol B	Japanese	ethanol	[47]
**111**	3′-geranylnaringenin	Japanese	ethanol	[47]
**103**	propolin A	Taiwanese glue	95% ethanol	[48]
**104**	propolin B	Taiwanese glue	95% ethanol	[48]
		Obtained by synthesis		
**112**	6-prenylnaringenin	synthesis		[12,14]
**113**	8-prenylnaringenin	synthesis		[12,14]
**114**	6,8-diprenylnaringenin	synthesis		[12,14]
**115**	6-geranylnaringenin	synthesis		[12,14]
**116**	8-geranylnaringenin	synthesis		[12,14]
**102**	isoxanthohumol	synthesis		[49]
**117**	4′-*O*-acylisoxanthohumol	synthesis		[49]
**118**	7,4′-*O*-diacetylisoxanthohumol	synthesis		[49]

**Table 5:** Chemical structures of flavanones monoprenylated in A-ring **84**–**89**, **91**–**93**, **99**, **100**–**102**, **106**, **107**, **112**, **113** and **115**–**118** with antioxidant activity.

N	Comp.	R^5^	R^6^	R^7^	R^8^	R^2′^	R^3′^	R^4′^
		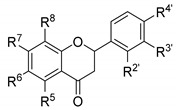						
**84**	5,7-dihydroxy-6-methyl-8-prenylflavanone	OH	Me	OH	prenyl	H	H	H
**85**	5,7-dihydroxy-4′-methoxy-6-methyl-8-prenylflavanone	OH	Me	OH	prenyl	H	H	OMe
**86**	5,7-dihydroxy-6-prenylflavanone	OH	prenyl	OH	H	H	H	H
**87**	5-hydroxy-7-methoxy-6-prenylflavanone	OH	prenyl	OMe	H	H	H	H
**88**	5,7-dihydroxy-4′-methoxy-8-prenylflavanone	OH	H	OH	prenyl	H	H	OMe
**89**	leachianone	OH	H	OH	prenyl	OH	H	OH
**91**	sophoraflavanone G	OH	H	OH	lavandulyl	OH	H	OH
**92**	kurarinone	OMe	H	OH	lavandulyl	OH	H	OH
**93**	kurarinol	OMe	H	OH	OHlavandulyl	OH	H	OH
**99**	isoglabranin	OH	prenyl	OH	H	H	H	H
**100**	glabranin	OH	H	OH	prenyl	H	H	H
**101**	7-methoxyisoglabranin	OH	prenyl	OMe	H	H	H	H
**102**	isoxanthohumol	OMe	H	OH	prenyl	H	H	OH
**106**	prokinawan	OH	HDMO ^1^	OH	H	H	OH	OH
**107**	nymphaeol A	OH	geranyl	OH	H	H	OH	OH
**112**	6-prenylnaringenin	OH	prenyl	OH	H	H	H	OH
**113**	8-prenylnaringenin	OH	H	OH	prenyl	H	H	OH
**115**	6-geranylnaringenin	OH	geranyl	OH	H	H	H	OH
**116**	8-geranylnaringenin	OH	H	OH	geranyl	H	H	OH
**117**	4′-*O*-acetylisoxanthohumol	OMe	H	OH	prenyl	H	H	OAc
**118**	7,4′-*O*-diacetylisoxanthohumol	OMe	H	OAc	prenyl	H	H	OAc

^1^ HDMO = 7-hydroxy-3,7-dimethyloct-2-enyl.

**Table 6:** Natural occurrence of prenylated isoflavones with antioxidant activity.

N	Trivial Name	Species	Extract	Ref.
		Fabaceae		
**119**	alpinum isoflavone	*Erythrina orientalis*	stem bark	[46]
**120**	8-prenyldaidzein	*Erythrina orientalis*	stem bark	[46]
**119**	alpinum isoflavone	*Erythrina stricta*	stem bark	[50]
**121**	erynone	*Erythrina stricta*	stem bark	[50]
**122**	wighteone	*Erythrina stricta*	stem bark	[50]
**123**	luteone	*Erythrina stricta*	stem bark	[50]
**119**	alpinum isoflavone	*Erythrina variegata*	stem bark	[51]
**124**	4′,5,7-trihydroxy-8-prenylisoflavone	*Erythrina variegata*	stem bark	[51]
		Moraceae		
**119**	alpinum isoflavone	*Ficus tikoua*	rhizomes	[52]
**125**	4′-*O*-methylalpinum isoflavone	*Ficus tikoua*	rhizomes	[52]
**126**	ficusin A	*Ficus tikoua*	rhizomes	[52]
**127**	ficusin C	*Ficus tikoua*	rhizomes	[52]
**128**	6-[(1R*,6R*)-3-methyl-6-(1-methylethenyl)-2-cyclohexen-1-yl]-5,7,4′-trihydroxyisoflavone	*Ficus tikoua*	rhizomes	[52]
		Apiaceae		
**119**	alpinum isoflavone	*Azorella madreporica*	aerial part	[53]
**129**	4′-*O*-acetylalpinum isoflavone	*Azorella madreporica*	aerial part	[53]
**130**	angustone C	*Azorella madreporica*	aerial part	[53]

**Table 7:** Natural occurrence of prenylated xanthone-type derivatives with antioxidant activity.

N	Trivial Name	Species	Extract	Ref.
		Moraceae		
**131**	cyclocommunol	*Artocarpus altilis*	heartwood and cortex	[35]
**132**	cycloartocarpin	*Artocarpus altilis*	heartwood and cortex	[35]
**133**	cyclogeracommunin	*Artocarpus altilis*	heartwood and cortex	[35]
**134**	isocycloartobiloxanthone	*Artocarpus altilis*	heartwood and cortex	[35]
**135**	cyclomorusin	*Artocarpus altilis*	heartwood and cortex	[35]
**136**	cudraflavone A	*Artocarpus altilis*	heartwood and cortex	[35]
**137**	artonin M	*Artocarpus altilis*	heartwood and cortex	[35]
**132**	cycloartocarpin	*Artocarpus anisophyllus*	heartwood and leaf	[18]
**138**	3′-hydroxycycloartocarpin	*Artocarpus anisophyllus*	heartwood and leaf	[18]
**139**	pyranocycloartobiloxanthone A	*Artocarpus anisophyllus*	heartwood and leaf	[18]
**133**	cyclogeracommunin	*Artocarpus communis*	cortex of root	[34]
**140**	cycloartobiloxanthone	*Artocarpus elasticus*	root bark	[34]
**141**	cycloartelastoxanthone	*Artocarpus elasticus*	root bark	[34]
**142**	artonol A	*Artocarpus elasticus*	root bark	[34]
**143**	cycloheterophyllin	*Artocarpus heterophyllus*	plant	[31]
**144**	artonin A	*Artocarpus heterophyllus*	plant	[31]
**145**	artonin B	*Artocarpus heterophyllus*	plant	[31]
**132**	cycloartocarpin	*Artocarpus incisa*	heartwood	[32]
**146**	tephrosin	*Artocarpus integer*	heartwood	[36]
**147**	artomandin	*Artocarpus kemando*	stem bark	[54]
**148**	artoindonesianin C	*Artocarpus kemando*	stem bark	[54]
**149**	artonol B	*Artocarpus kemando*	stem bark	[54]
**140**	cycloartobiloxanthone	*Artocarpus nobilis*	root bark	[33]
**150**	artobiloxanthone	*Artocarpus nobilis*	root bark	[33]
**139**	pyranocycloartobiloxanthone A	*Artocarpus obtusus*	stem bark	[55]
**136**	cudraflavone A	*Artocarpus scortechinii*	leaf and stem bark	[19]
**140**	cycloartobiloxanthone	*Artocarpus scortechinii*	leaf and stem bark	[19]
